# Late Pleistocene human paleoecology in the highland savanna ecosystem of mainland Southeast Asia

**DOI:** 10.1038/s41598-021-96260-4

**Published:** 2021-08-18

**Authors:** Kantapon Suraprasit, Rasmi Shoocongdej, Kanoknart Chintakanon, Hervé Bocherens

**Affiliations:** 1 Morphology of Earth Surface and Advanced Geohazards in Southeast Asia Research Unit (MESA RU), Department of Geology, Faculty of Science, Chulalongkorn University, Bangkok, 10330 Thailand; 2 Department of Geosciences, Biogeology, University of Tübingen, Hölderlinstraße 12, 72074 Tübingen, Germany; 3 Department of Archaeology, Faculty of Archaeology, Silpakorn University, Bangkok, 10200 Thailand; 4 The Prehistoric Population and Cultural Dynamics in Highland Pang Mapha Project, Princess Maha Chakri Sirindhorn Anthropology Centre, Bangkok, 10170 Thailand; 5 Senckenberg Research Centre for Human Evolution and Palaeoenvironment (S-HEP), University of Tübingen, Sigwartstraße 10, 72076 Tübingen, Germany

**Keywords:** Ecology, Evolution, Biogeochemistry

## Abstract

The late Pleistocene settlement of highland settings in mainland Southeast Asia by *Homo sapiens* has challenged our species’s ability to occupy mountainous landscapes that acted as physical barriers to the expansion into lower-latitude Sunda islands during sea-level lowstands. Tham Lod Rockshelter in highland Pang Mapha (northwestern Thailand), dated between 34,000 and 12,000 years ago, has yielded evidence of Hoabinhian lithic assemblages and natural resource use by hunter-gatherer societies. To understand the process of early settlements of highland areas, we measured stable carbon and oxygen isotope compositions of Tham Lod human and faunal tooth enamel. Our assessment of the stable carbon isotope results suggests long-term opportunistic behavior among hunter-gatherers in foraging on a variety of food items in a mosaic environment and/or inhabiting an open forest edge during the terminal Pleistocene. This study reinforces the higher-latitude and -altitude extension of a forest-grassland mosaic ecosystem or savanna corridor (farther north into northwestern Thailand), which facilitated the dispersal of hunter-gatherers across mountainous areas and possibly allowed for consistency in a human subsistence strategy and Hoabinhian technology in the highlands of mainland Southeast Asia over a 20,000-year span near the end of the Pleistocene.

## Introduction

**Figure 1:**
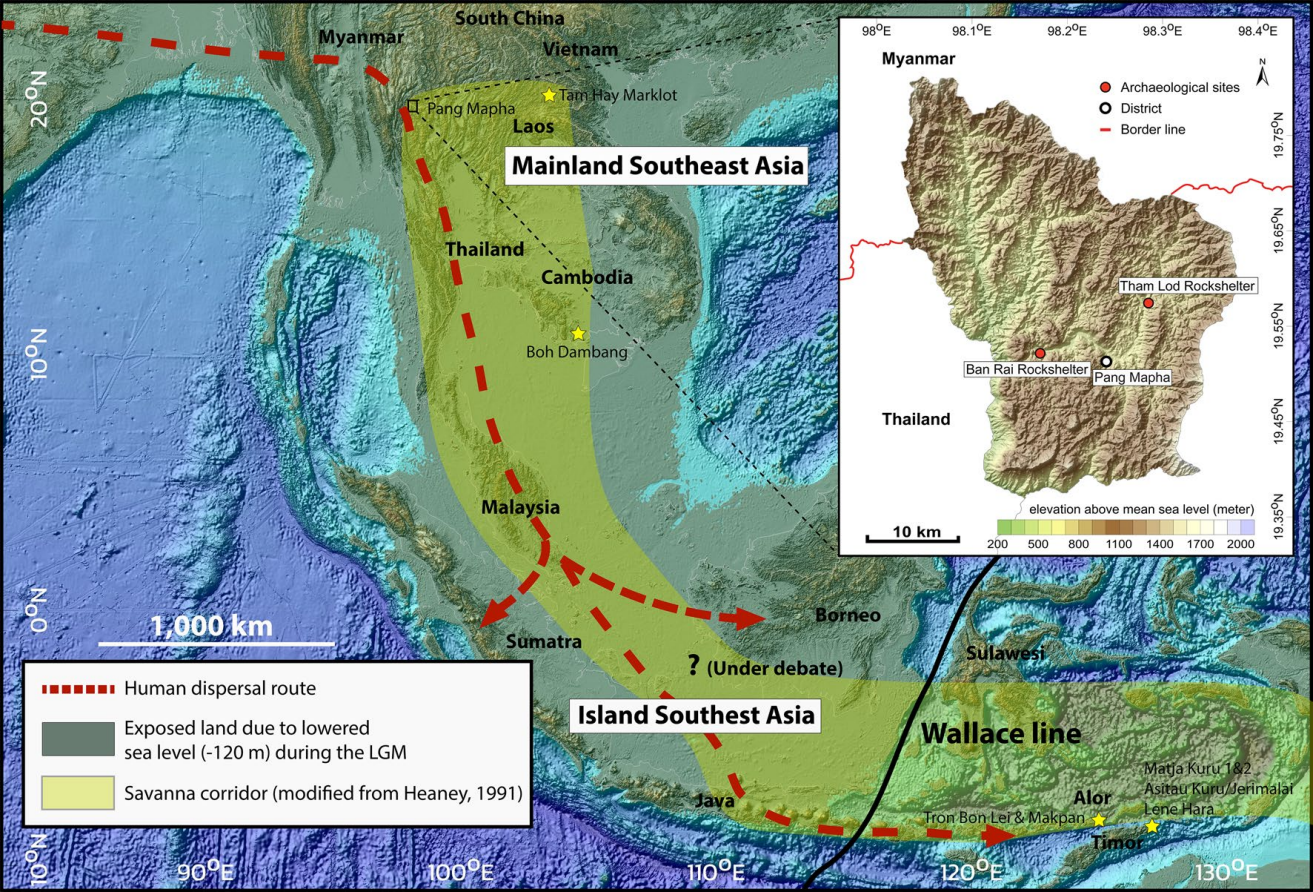
Map of Southeast Asia showing the location of archaeological sites in Pang Mapha, Mae Hong Son Province, northwestern Thailand and indicating the dispersal route of early modern humans (red dashed line, modified from^7^) and the extension area of a hypothesized north–south savanna corridor (yellow area) during the LGM (modified from^2^). The continuity of the band of open vegetation existing along the transequatorial region of central Sundaland remains under debate (e.g.,^4,8–10^) (see^4,6,10^ for other detailed information and available proxy data regarding the LGM vegetation cover across Southeast Asia). Stars indicate the location of late Pleistocene fossil sites where stable isotope analyses of mammal tooth enamel have been performed. The map was generated in Surfur software (version 11, https://www.goldensoftware.com) and the figure was created using Adobe Illustrator CS5 (https://www.adobe.com).

Our species has spread worldwide and successfully occupied a diversity of extreme environments such as deserts, arctic environments, tropical rainforests, and high-altitude conditions during the late Pleistocene (126–12 ka) (^1^). During the process of dispersal of *Homo sapiens* out of Africa to ultimately reach Australia, mainland Southeast Asia (MSEA) is recognized as being a potential route of human migration prior to crossing through the Sunda Shelf during a period of glaciation (Fig. 1). In terms of paleoenvironments, much botanical, biogeographical, and geochemical evidence has suggested that a north–south savanna corridor (i.e. a band of open vegetation or a mixture of forest/grassland ecosystem) was present, starting from the central part of Thailand and streching across the exposed Sunda shelf during the Last Glacial Maximum (LGM, around 29–17 ka) when the sea level dropped up to 120 m below the present-day stand (e.g.,^2–5^). Such a corridor might have facilitated the rapid dispersal of early humans through the intermediate migratory region of SEA^4,6^. However, the range expansion of inland vegetation examining the northern limit of a savanna corridor during the LGM is not yet known in great detail and the past human–environment interactions in the highlands of MSEA remain barely understood. Paleoecological and paleoenvironmental studies of the late Pleistocene hominin-bearing faunas in MSEA are thus important to understand the early modern human mobility and migration patterns through the land corridor into island Southeast Asia (ISEA).

**Figure 2:**
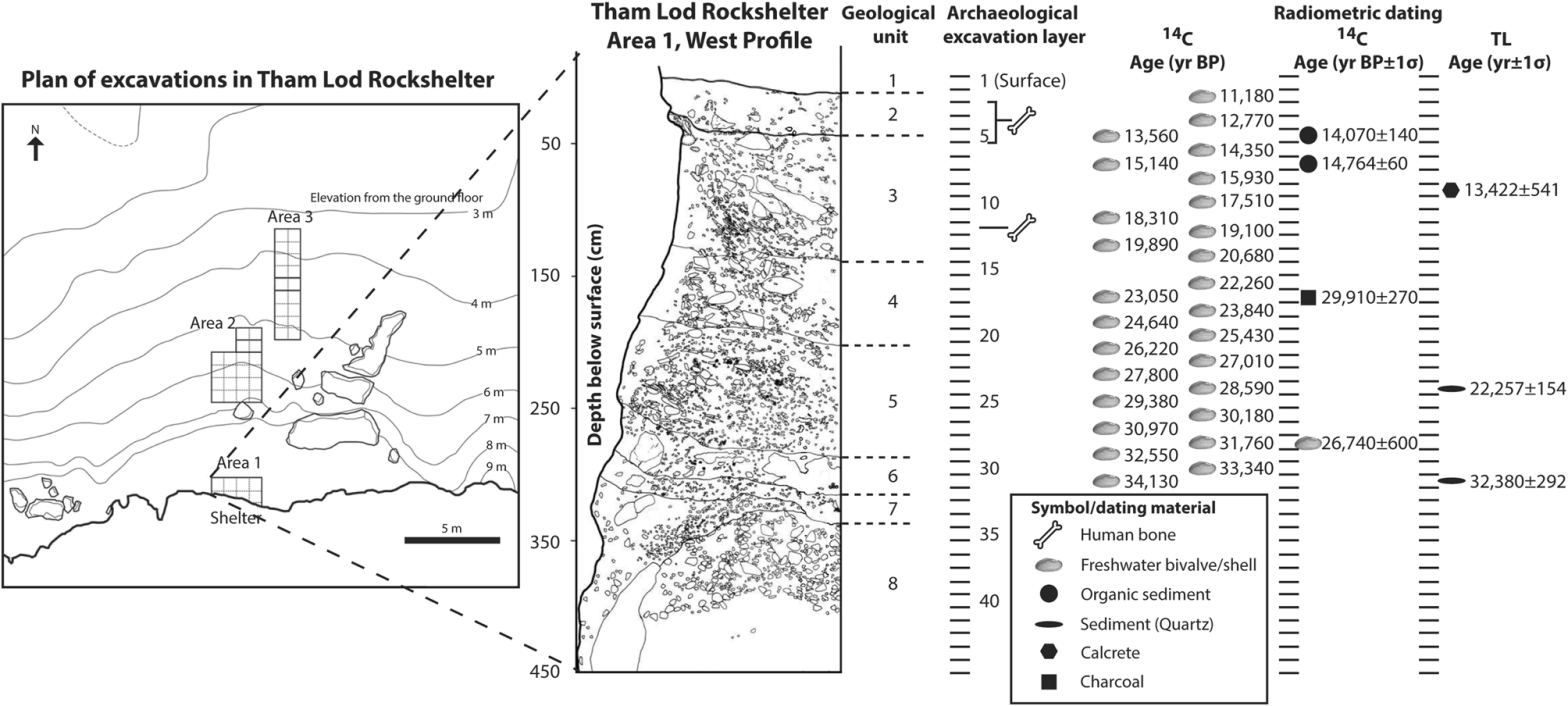
A plan of excavations and stratigraphic profile of the Area 1 of Tham Lod Rockshelter. Ages of each stratigraphic layer obtained from different dating methods and material^14,15,28,29^ are plotted together. In this study, all isotope samples of humans and mammals were collected from the layer 1 (top) to 31 (bottom) of the TLR Area 1. The figure was created using Adobe Illustrator CS5 (https://www.adobe.com).

Mainland Southeast Asia has yielded a high number of archaeological sites with rich animal remains and lithic artifacts, although human remains were rarely found in association with them especially during the late Pleistocene (e.g.,^11–19^). The earliest modern human fossils, dated to a minimum age of 63–43 ka, were recovered from Tam Pa Ling, up to 1170 m in elevation, in Laos^16,17^. The increasing number of archaeological records in Thailand and Laos also supports the idea that high-altitude karst settings such as caves and rockshelters (e.g., Spirit Cave, Tham Lod Rockshelter, Ban Rai Rockshelter, Doi Pha Kan, and Pha Phen) were often used by hunter-gatherers^14,15,20–23^. Chrono-cultural frameworks for human remains and stone artifacts recovered from many archaeological sites in MSEA have suggested a typical “Hoabinhian” techno-complex characterized by flexed burials, sumatraliths, large and small tools made on cobbles, with an age ranging from the late Pleistocene to the mid-Holocene (e.g.^23–27^). A highland area (a range of low mountains or elevated parts of the country) is topographically characteristic of northern Thailand, which shares borders with Myanmar and Laos. Tham Lod Rockshelter (TLR) in Pang Mapha District (Mae Hong Son Province, northwestern Thailand) situated on a mountainous karst landscape with the elevation of 640 m above mean sea level (Fig. 1) is a good example of such important highland sites where the human and animal remains associated with the Hoabinhian techno-complex have systematically been excavated with available and detailed stratigraphic, taphonomic, zooarchaeological, and chronological data^14,15,26,28–31^. Radiocarbon (^14^C) and thermoluminescence (TL) dating methods on various material including charcoal, sediments, and freshwater shells collected along the stratigraphic section of TLR have provided measurements for the faunal age ranging from 34 to 12 ka^14,15,28,29^ (Fig. 2 and see Supplementary Information 1 and Supplementary Tables S1 and S2 for more detailed information on geological, dating, faunal, and zooarchaeological contexts). The rockshelter was initially occupied by hunter-gathers since 34 ka^14,15^, while the human skeletal remains were recovered only from the upper part of the TLR stratigraphic sequence (layers 3, 4, 5, and 12 in the Area 1), dated between 19 and 12 ka (Fig. 2). The zooarchaeological analyses of a mammalian assemblage and the detailed studies of lithic assemblages in TLR have provided some information about subsistence patterns of hunter-gatherers in the area^26,29–31^. However, based on these approaches alone, it is difficult to describe properly the ecological niche of late Pleistocene hunter-gatherer populations and to refine the relationship between lithic technology and paleoenvironments in the highland of MSEA. More direct investigations of human resource reliance, using other multidisciplinary methods, are thus required.

To understand resource-exploitation strategies of early humans, an isotopic approach applied to faunal fossilized tissues has proven to be useful in reconstructing the diet and habitat of ancient mammals as well as the paleoenvironmental and paleoclimatic contexts for human occupations (e.g.,^32–35^). Although carbon and nitrogen isotope measurements of dentine collagen of a few late Pleistocene mammals (38.4–13.5 ka) from the cave of Tam Hay Marklot in northern Laos have successfully been carried out^36^, stable isotope tracking on dentine or bone collagen of human and animal remains from several archaeological sites in MSEA has been rather limited likely due to their location under the tropical monsoon climate where warm temperature and humidity usually prevented the good preservation of biological molecules such as DNA and collagen (e.g.,^37,38^). In the case of TLR, faunal remains exhibited poor collagen preservation as indicated by the low amounts of nitrogen content (using a CNS analysis) of several dentine and bone samples (Supplementary Tables S3). Alternatively, measurements of stable carbon and oxygen isotope ratios in tooth enamel are one of the most efficient tools available to address this issue. So far, this approach has never been applied directly to the paleoecological study of any human-bearing faunas with this age range in MSEA.

Here we performed stable carbon and oxygen isotope analyses of human and faunal tooth enamel collected from the Area 1 of TLR (Fig. 2). The aim of this study is to investigate the ecological niche of late Pleistocene hunter-gatherer populations and their associated mammal assemblages and to determine the role of faunal resources and local environments on human exploitation strategies in the region. In the light of stable isotope data, we propose an environmental scenario for the process of early modern human settlements of highland SEA with implications for the dispersal of *Homo sapiens* across MSEA during the LGM.

## Results

### Isotope analysis of tooth enamel

The CaCO_3_ content of all TLR enamel samples (n = 195) varies from 2.0 to 6.9% and falls mostly within the normal range of recent samples that do not show evidence of diagenetic effects (e.g.,^39^) (see Supplementary Table S4 for a stable isotope dataset). Although some high values may reflect a minor contribution of isotopic exchanges with exogenous sedimentary carbonates, there is no correlation between CaCO_3_ content and *δ*^13^C and *δ*^18^O values for those samples (Supplementary Fig. S1). Therefore, there is no indication that the pretreated enamel samples have not yielded biogenic isotope signals.

Carbon isotope results are interpreted based on estimated cut-off values for diets of C_3_ *versus* C_4_ (grasses or sedges) plants and for habitats of closed *versus* open canopies. In the humid tropics, the cut-off *δ*^13^C values of − 10‰ and − 2‰ are applied for the dietary category distinction between herbivorous ungulates (< − 10‰ for a pure C_3_ diet, between − 10‰ and − 2‰ for a mixed C_3_/C_4_ diet, and >  − 2‰ for a pure C_4_ diet)^34,40,41^. In terms of habitats, the cut-off *δ*^13^C values lower than − 13‰ refer to the browsers that occupied a closed forest canopy, while the cut-off *δ*^13^C values higher than − 10‰ indicate a mixed feeder and/or grazer in more open habitats (e.g.,^35,41^). In terms of trophic relationships, all *δ*^13^C values of predators present in this study (i.e. *Panthera tigris*) are adjusted upward by + 1.3‰ for a direct comparison with those of herbivores^42,43^). In omnivores such as some primates (including humans), rodents (i.e. *Hystrix*), and ursids (i.e. *Ursus thibetanus*), stable carbon isotopes in tooth enamel are incapable of distinguishing between plant- and meat-based (or insect-based) diets and the carbon isotopic fractionation between their food and tooth enamel remains unclear. In this study, we keep reporting enamel *δ*^13^C values of omnivores without isotopic fractionation corrections, which may not allow us to compare directly the trophic relationships between omnivores and herbivores/carnivores. The isotopic enrichment of ^13^C between diet and bioapatite in omnivorous mammals varies among species likely due to differences in their body sizes and physiological, anatomical, and behavioural traits (e.g.,^44,45^). However, the *δ*^13^C values of omnivores can reflect the dietary intake of the ultimate plant resources (C_3_ or C_4_)^46^.

The *δ*^18^O carbonate of tooth enamel is commonly used to investigate the isotopic composition of ingested water, despite having been influenced by several important factors (e.g., latitude, altitude, aridity, precipitation, and evaporation). As the oxygen isotopic fractionation of meteoric water is incorporated into the animal's tissues via obligate drinking and/or plant-derived intake, some principles are often applied to the study of fossil mammals within the same temporal and spatial coverage. Mammals frequently ingesting water through drinking are expected to have lower *δ*^18^O enamel values than those of drought-tolerant taxa^47,48^. Grassland-inhabiting grazers may have higher *δ*^18^O enamel values than forest-dwelling browsers^35,49^.

### Bulk isotope analysis

**Figure 3:**
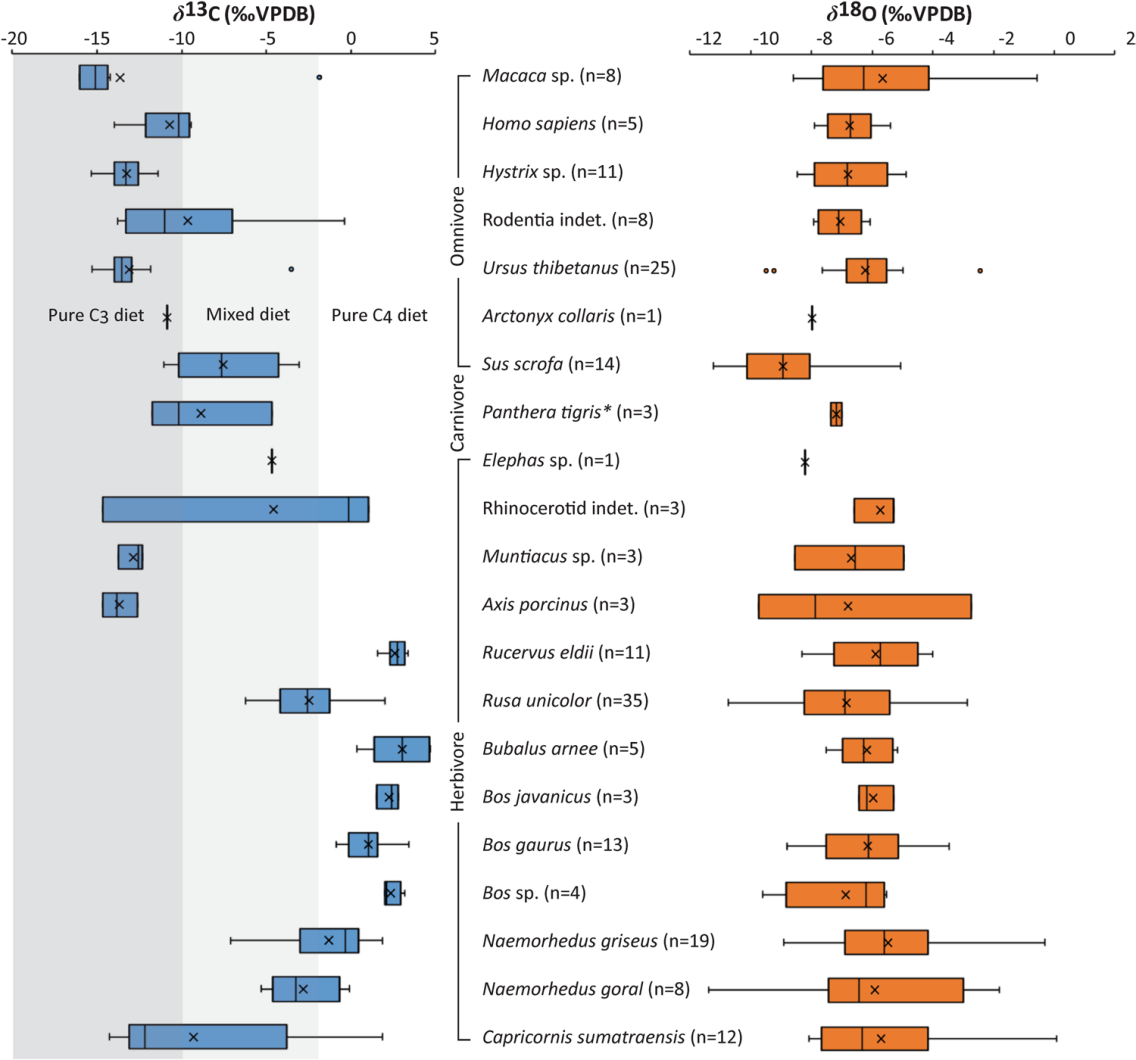
Box plots of bulk *δ*^13^C (blue) and *δ*^18^O (orange) values of human and faunal tooth enamel from the late Pleistocene of Tham Lod Rockshelter in highland Pang Mapha, northwestern Thailand. Asterisks (*) indicate carnivorous mammals with adjusted *δ*^13^C values of + 1.3‰ according to^42,43^.

All the *δ*^13^C values of human and mammal tooth enamel displayed a median of − 4.3‰ and ranged from − 16.0‰ to + 4.7‰, indicating reliance on a broad range of ecosystems expanding from pure C_3_ to C_4_ vegetation and corresponding to dense forests to open environments (Fig. 3). The enamel carbonate *δ*^18^O values of all human and faunal samples exhibited a median of − 6.6‰ and ranged from − 11.4‰ to + 0.1‰. Statistically examined by the Kruskal–Wallis tests, there are significant differences in median *δ*^13^C and *δ*^18^O values between the investigated mammalian groups (*δ*^13^C: H = 138.80, *P* < 0.01 and *δ*^18^O: H = 32.51, *P* < 0.01, n = 174) (see Supplementary Table S5 for Mann–Whitney pairwise comparisons between taxa).

#### ***δ***^13^C

##### Omnivore

*Primates* The *δ*^13^C values of *Macaca* sp. (median: − 15.1‰, range: − 16.0‰ to − 1.9‰, n = 8) suggested the consumption of pure C_3_-based resources in dense forest environments. One peculiar sample with a C_4_ signal (− 1.9‰) suggests that some individuals were able to consume grasses or possibly ate chiefly grass-eating insects such as grasshoppers, as observed in the feeding habits of extant long-tailed macaques in Malaysia and Java^50,51^.

*Humans* The *δ*^13^C values of human teeth (median: − 10.2‰, range: − 14‰ to − 9.4‰, n = 5) almost suggested the consumption of a combination between the higher amount of C_3_ foods (plants and/or C_3_ plant-dwelling preys) and some C_4_ items and/or the occupation in open forests (more open habitats compared to *Macaca* sp., *P* < 0.05). The most negative *δ*^13^C value of − 14‰ in the tooth sample collected from the layer 3 near the ground surface showed human reliance on pure C_3_-based resources in a closed forest canopy.

*Rodents* The *δ*^13^C values of *Hystrix* sp. (median: − 13.3‰, range: − 15.3 to − 11.4‰, n = 11) suggest that porcupines consumed only pure C_3_-based resources in closed or open canopy forests, which were not as dense as where the macaques occupied (*P* < 0.05). The *δ*^13^C values of small indeterminate rodents (median: − 11.0‰, range: − 13.8‰ to − 0.4‰, n = 8) yielded a high variability in dietary intake and habitat use ranging from closed C_3_ to open C_4_ ecosystems (*P* > 0.05 between indeterminate rodents and humans).

*Omnivorous Carnivora Ursus thibetanus* (median: − 13.5‰, range: − 15.3‰ to − 3.5‰, n = 25) might have had the primary diet of C_3_ food items (e.g., fruits from trees as well as insects, invertebrates, and small vertebrates that ate C_3_ plants) similar to that of extant populations^52^, and shared a closed habitat with the porcupines (*Hystrix* sp.) (*P* > 0.05) and some ruminants such as small-sized deer *Muntiacus* sp. and *Axis porcinus*. One sample of *Arctonyx collaris* (− 10.9‰) reflected its food preferences on C_3_ items such as tubers, roots, and small creatures^52^ and its occupation of an open forest landscape.

*Suids* Samples of *Sus scrofa* (median: − 7.6‰, range: − 11.1‰ to − 3.1‰, n = 14) imply that their diet consisted of a mixture of C_3_ and C_4_ resources in an intermediate area between open and closed landscapes or sometimes had a pure C_3_ diet in an open forest. This also indicates that both types of vegetation were available at the same time and in a limited area.

##### Carnivore

*Predatory Carnivora* The adjusted *δ*^13^C values of *Panthera tigris* (median: − 10.2‰, range: − 11.7‰ and − 4.7‰, n = 3) suggest that tigers were probably preying essentially on *S*. *scrofa* or might have been consumers of a wide range of prey species in both open and closed canopies.

##### Herbivore

*Elephants* One sample of *Elephas* sp. exhibited a *δ*^13^C value of − 4.7‰, which suggested a mixture of C_3_ and C_4_ plants in an intermediate area between closed and open canopy landscapes.

*Rhinoceroses* The rhinocerotid enamel samples (median: − 0.1‰, range: − 14.6‰ to + 1.0‰, n = 3) suggested a wide range of diets varying from pure C_3_ to C_4_ vegetation. Otherwise, it is possible that there were two separate rhinocerotid taxa in the locality, one C_3_ browsing species (e.g., *Rhinoceros sondaicus*) and two C_4_ grazing individuals (e.g., *Rhinoceros unicornis*), as observed in some Pleistocene Southeast Asian sites^53^.

*Cervids* The small-sized muntjac deer, *Muntiacus* sp. (median: − 12.6‰, range: − 13.7‰ to − 12.3‰, n = 3), had pure C_3_-plant diets and occupied a forest landscape, similar to the extant population of *Muntiacus muntjak* (Indian muntjac)^52^. The samples of *Axis porcinus* (median: − 13.8‰, range: − 14.7‰ to − 12.6‰, n = 3) indicated a pure C_3_ plant diet in more closed canopy habitats, compared to *Muntiacus* sp. The Eld's deer *Rucervus eldii* (median: + 2.7‰, range: + 1.5‰ to + 3.4‰, n = 11) had a pure C_4_ diet and occupied an open habitat. The sambar deer *Rusa unicolor* (median: − 2.6‰, range: − 6.2‰ to + 2.0‰, n = 35) indicated their primary utilization of mixed C_3_/C_4_ plants but sometimes pure C_4_ ones and had the occupation of open canopy habitats but more closed than where *Rucervus eldii* lived (*P* < 0.05). Unlike their modern representatives that have a habitat shift to more closed forests^10,52^, the late Pleistocene *Rucervus eldii* and *Rusa unicolor* in TLR were reliant on an open grassland landscape. It is apparent that substantial resource partitioning and minimized intergeneric competition occurred among TLR cervid communities, leading to the prevalent coexistence of four cervid genera/species during the late Pleistocene.

*Bovines* Samples of wild water buffaloes, *Bubalus arnee*, (median: + 3.0‰, range: + 0.3‰ to + 4.7‰, n = 5) reflected a pure C_4_-plant diet. Two large bovid species, *Bos javanicus* (median: + 2.4‰, range: + 1.5‰ to + 2.8‰, n = 3) and *Bos gaurus* (median: + 1.0‰, range: − 0.9‰ to + 3.4‰, n = 13), consumed substantial amounts of C_4_ plants, similar to *Bubalus arnee* (*P* > 0.05). The samples of undetermined species, *Bos* sp. (median: + 2.1‰, range: + 2.0‰ to + 3.2‰, n = 4) also focused on pure C_4_ vegetation in an open habitat.

*Caprines* Stable isotope data of two goral taxa (Chinese goral *Naemorhedus griseus* and Himalayan goral *Naemorhedus goral*) and one serow species (sumatran serow *Capricornis sumatraensis*) from TLR were analyzed by^54^. Both *Naemorhedus griseus* (median: − 0.4‰, range: − 7.1‰ to + 1.9‰, n = 19) and *Naemorhedus goral* (median: − 3.3‰, range: − 5.3‰ to − 0.1‰, n = 8) showed the same dietary habits in foraging on mixed C_3_ and C_4_ vegetation and on pure C_4_ plants (*P* = 0.05)^54^. However, *Capricornis sumatraensis* (median: − 12.2‰, range: − 14.3‰ to + 1.9‰, n = 12) had a wider range of diets, compared to the two goral species (*P* < 0.05), reflecting its occupation of closed C_3_- to open C_4_-dominated landscapes^54^.

#### ***δ***^18^O

Several samples from the same taxa/locality showed a high variability and relatively wide ranges of *δ*^18^O values (Fig. 3) that likely resulted from the influence of several important controlling factors such as the isotopic composition of ingested water, the consistent fractionation of oxygen isotopes between body water and tooth enamel, and the animal's metabolism (e.g.,^55,56^) or from the nonsimultaneous occurrence of fossils that represented a different age through the stratigraphic section. The pairwise Mann–whitney U test indicates that there are almost no statistically significant differences in median *δ*^18^O values between two TLR mammal taxa (*P* > 0.05), except for wild boars (Supplementary Table S5). The wild boars displayed lower *δ*^18^O values (median: − 8.9‰, range: − 11.2‰ to − 5.1‰, n = 14), compared to other mammal taxa within the same locality (*P* < 0.05). It is possible that these suids fed on more ^18^O- depleted foodstuffs (such as fallen fruits, roots, and tubers) on the open forest/woodland ground surfaces^57,58^.

### Serial sampling isotope analysis

**Figure 4:**
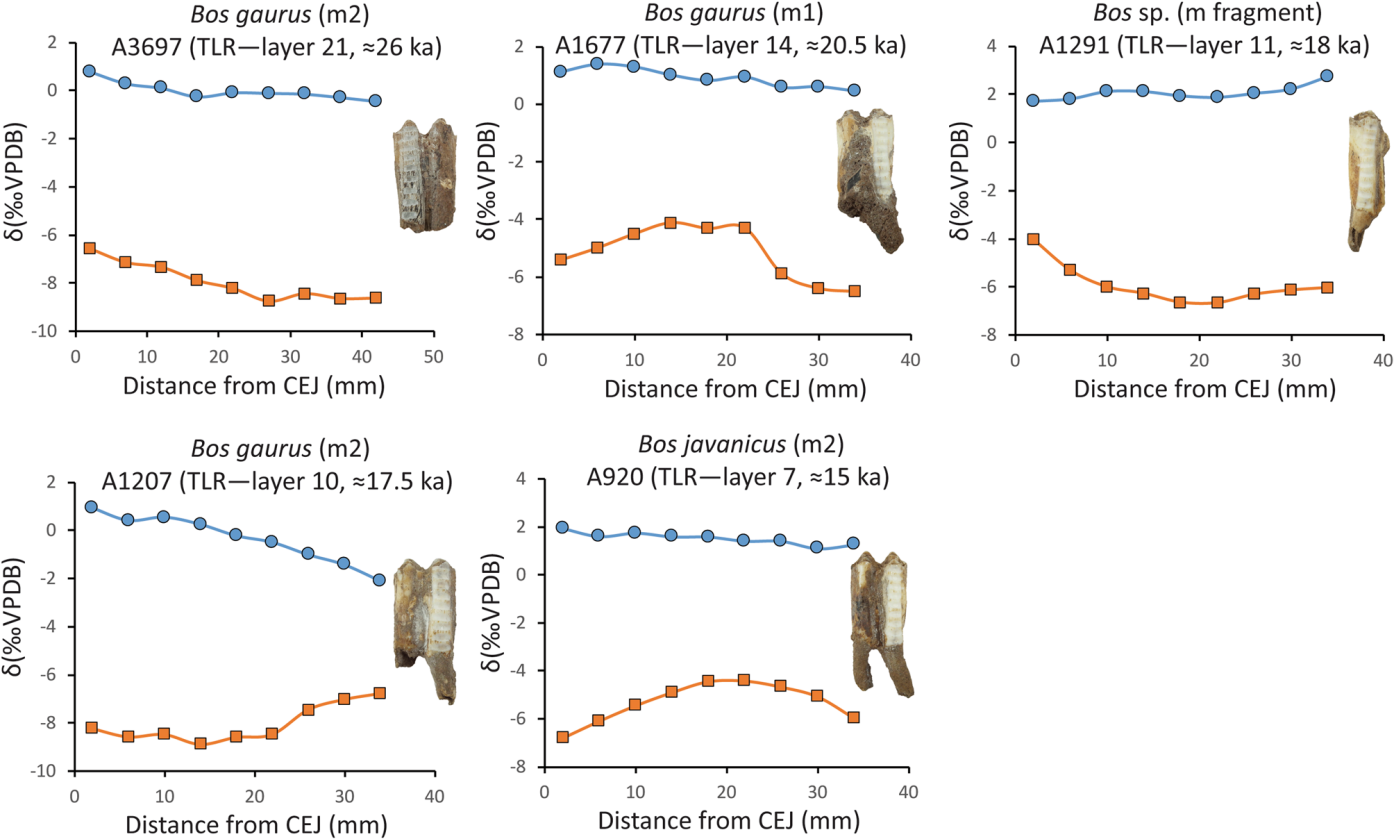
Sequential tooth enamel *δ*^13^C (blue circle) and *δ*^18^O (orange square) records along the crown height of large bovids. *TLR* (Tham Lod Rockshelter) and *CEJ* (Cemento-enamel junction).

As large bovid molars gradually mineralize on average with the enamel growth rate of approximate 40–50 mm (in height) per year^59–61^, we analyzed five high-crowned molars of large bovids (*Bos gaurus* (n = 3), *Bos javanicus* (n = 1), and *Bos* sp. (n = 1)) from different stratigraphic layers of TLR to demonstrate seasonal patterns in diet and precipitation through the late Pleistocene (Fig. 4 and Supplementary Table S6).

Our serially analyzed isotope samples of large bovid molars showed little *δ*^13^C variation of 1.2‰ (from − 0.4‰ to + 0.8‰) for TLR-A3697, 0.9‰ (from + 0.5‰ to + 1.4‰) for TLR-A1677, 1.0‰ (from + 1.7‰ to + 2.7‰) for TLR-A1291, 3.0‰ (from − 2.1‰ to + 0.9‰) for TLR-A1207, and 0.8‰ (from + 1.1‰ to + 1.9‰) for TLR-A920, all of which indicate grazing habits on the same types of food items (pure C_4_ plants) throughout the course of almost 1 year during the terminal Pleistocene (Fig. 4).

The serial *δ*^18^O values of large bovids yielded a small fluctuation of 2.2‰ (from − 8.8‰ to − 6.6‰) for TLR-A3697, 2.4‰ (from − 6.5‰ to − 4.1‰) for TLR-A1677, 2.7‰ (from − 6.7‰ to − 4.0‰) for TLR-A1291, 2.1‰ (from − 8.9‰ to − 6.8‰) for TLR-A1207, and 2.4‰ (from − 4.4‰ to − 6.8‰) for TLR-A920. All the serially analyzed samples showed little seasonal variation in precipitation over several months to years through the terminal Pleistocene (Fig. 4).

## Discussion

### Expansion of savanna corridor and environmental/climatic impacts on hunter-gatherer settlement

**Figure 5:**
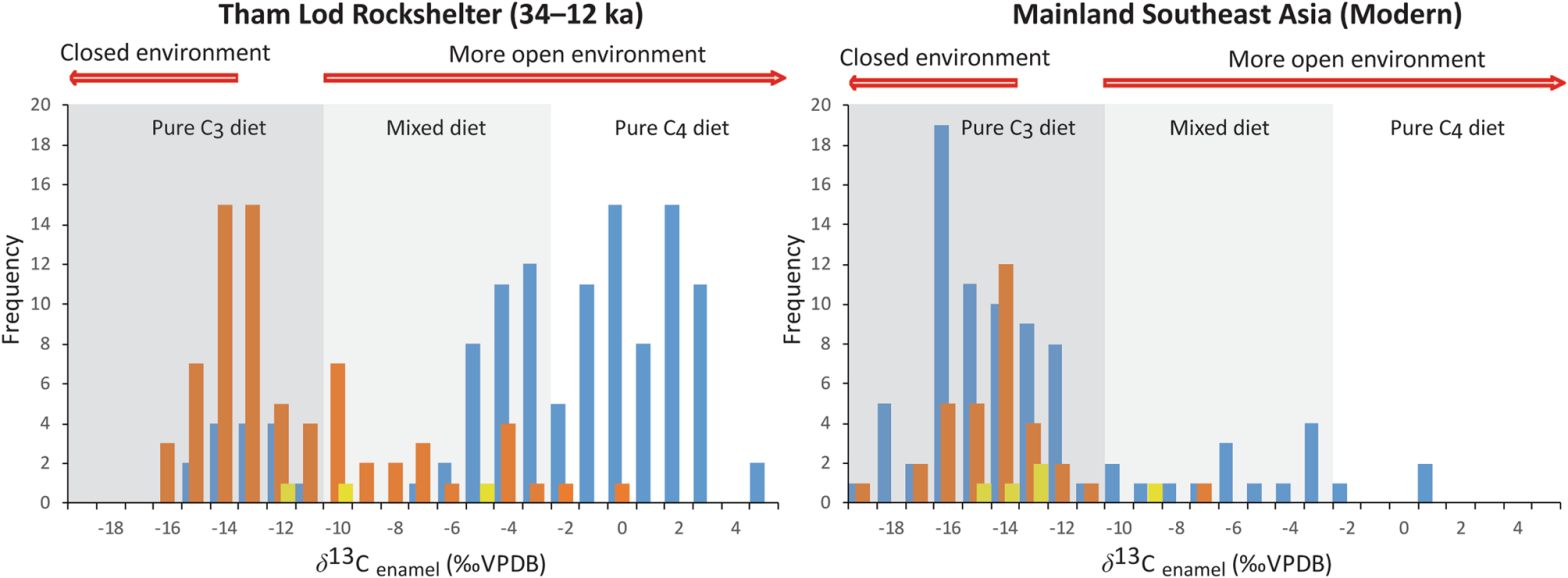
Distribution of enamel *δ*^13^C values of mammalian assemblages (herbivores (blue), omnivores (orange), and carnivores (yellow)) between Tham Lod Rockshelter (terminal Pleistocene, n = 195) and mainland Southeast Asia (modern samples, n = 121). Stable carbon isotope datasets for modern mainland Southeast Asian mammals were obtained from the existing literature^10,54,63,64^. A Suess effect *δ*^13^C correction is applied for samples of modern mammals that died after ad 1950^34,65^. The samples of carnivorous mammals were adjusted by + 1.3‰^42,43^. All enamel *δ*^13^C values plotted here are given in Supplementary Tables S4 and S7.

Our carbon isotope results of the extensive faunal baseline implied the expansion of a forest-grassland mosaic ecosystem into the high-altitude or mountainous area of Pang Mapha, up to about 600 m above present-day sea level, where the mixture of semi-evergreen and dry dipterocarp forests is typical of this elevation today^14^. Many types of savanna formations are still present today across MSEA, but they are mostly patchy and fragmented^62^. This study suggests that mixed tropical forest/grasslands were more widespread and connected in MSEA during the terminal Pleistocene, as many grazing species relied exclusively on C_4_ grasses (Fig. 5 and see^4,10^ for more detailed information and other available proxy sources). As supported by similar isotope compositions of a contemporaneous mammal fauna from the slightly higher-latitude cave of Tam Hay Marklot in Laos^36^, we purport to document the possible extension of a latitudinal limit of the savanna corridor farther north than previously recorded during the LGM (Fig. 1). Despite the hunter-gatherers having faced with the landscape of high mountain ranges in the northern part of MSEA during the process of dispersal, such a savanna corridor widely stretching from northern Thailand and Laos to either Central Sundaland or Western Java would have served as a convenient route for early human and large mammal migrations out of MSEA, across the Sunda Shelf, and into ISEA during the LGM.

**Figure 6:**
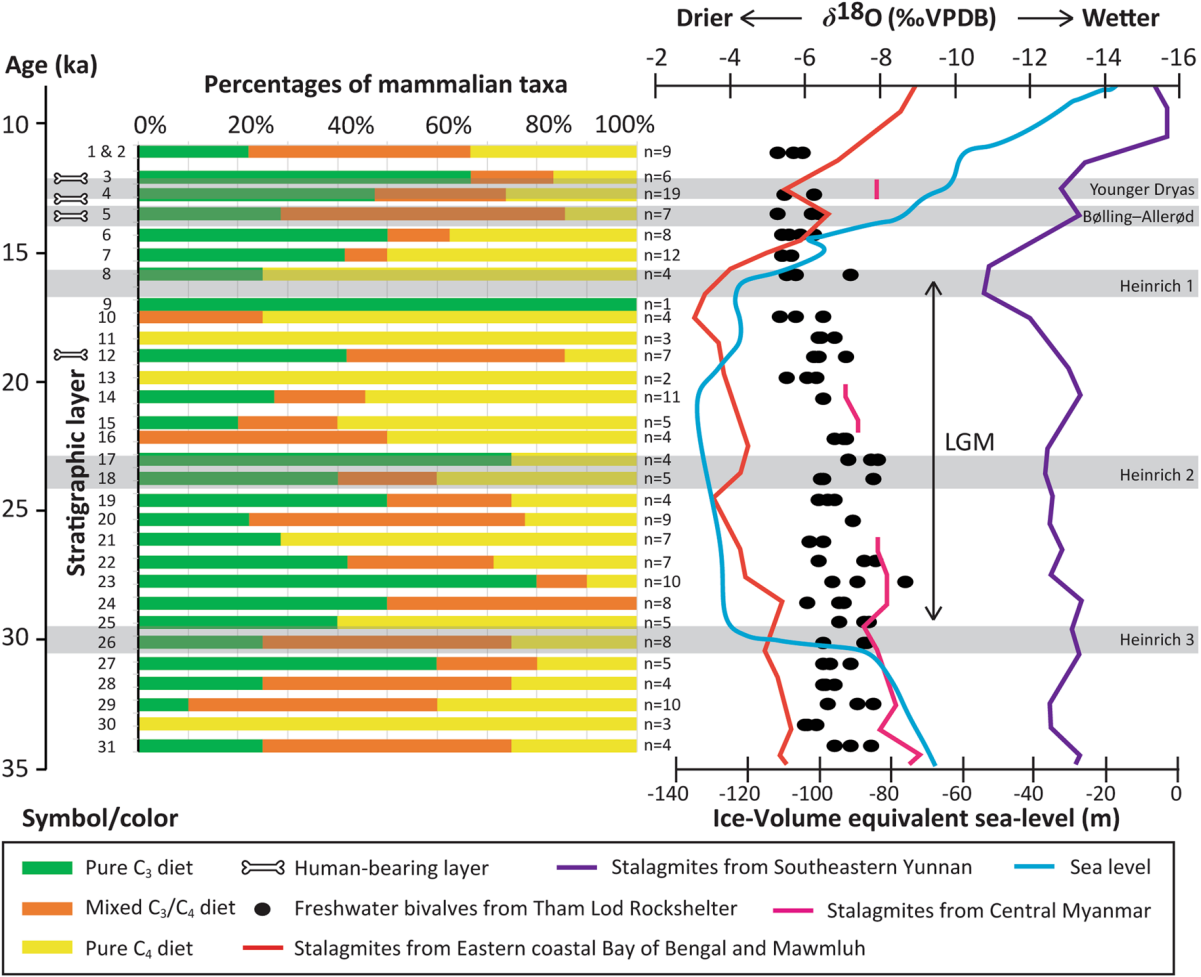
Trends of ecosystems over a time period of the terminal Pleistocene (34–12 ka) based on percentages of C_3_ browsers (forests/woodlands), C_3_–C_4_ mixed feeders (intermediate landscapes between closed and open canopies), and C_4_ grazers (grasslands) through the stratigraphic sequence of Tham Rod Rockshelter, in comparison with freshwater bivalve *δ*^18^O data (black dot) collected from the same stratigraphic section (west profile)^28^, with speleothem *δ*^18^O records data using 1000-year averages from mainland Southeast Asia including eastern coastal Bay of Bengal and Mawmluh (red line), central Myanmar (pink line), and southeastern Yunnan (purple line)^66–68^, and with sea-level fluctuations (sky blue)^69^. Available dietary information for all mammalian taxa from TLR was distinguished based on the carbon isotope data with the cut-off *δ*^13^C values lower than − 10‰ for a pure C_3_ diet, between − 10‰ and − 2‰ for a mixed C_3_/C_4_ diet, and higher than − 2‰ for a pure C_4_ diet^34,40,41^. High-resolution chronological data of the TLR sequence follow radiobarbon dates on freshwater bivalves collected from each stratigraphic layer^28^.

Although no significant shift in *δ*^13^C and *δ*^18^O values among mammalian individuals through the stratigraphic section of TLR (Fig. 6 and Supplementary Figs. S2 and S3) and no major seasonal variation in dietary and climatic patterns acquired from the intra-tooth profiles of large bovid crowns over the year (Fig. 4) are discernible through this study, other related information and proxies have provided direct evidence for the regional climate changes through the period of late Pleistocene glaciation. Sequential *δ*^18^O records of analyzed freshwater bivalves collected from TLR implied wetter and relatively unstable climatic conditions from 35 to 20 ka and drier conditions from 20 to 11.5 ka (with an aridity peak around 15.6 ka)^28^ (Fig. 6). Speleothem *δ*^18^O records obtained from nearby regions (western Thailand, Myanmar, and South China) indicated a dramatic shift in precipitation to the wetter climate condition starting around 16 ka (Heinrich event 1) and at the end of the Pleistocene (Younger Dryas)^66–68^ (Fig. 6).

As demonstrated by our carbon isotope data through the stratigraphic sequence of TLR, it seems unlikely that the Bølling–Allerød event affected human subsistence patterns at that period due to the persistence of heterogeneous environments with the availability of potential needed resources (Fig. 6). Although the homogeneity of environments has been discernible through some stratigraphic layers (i.e. layers 9, 11, 13, and 30) of TLR (Fig. 6), this was most likely due to the effects of analyzing an isotope dataset with small sample sizes. In relation to sea-level rise during the terminal Pleistocene-Holocene boundary^69^, the broad expansion of tropical rainforests or the replacement of forest/grassland mosaics by closed forest environments has also been documented elsewhere based on several studies and proxy records in SEA^70^. One human sample (specimen no. 611) collected at the layer 3 of TLR (dated around 12 ka) showed alongside a shift in *δ*^13^C towards higher reliance on C_3_-based resources in a more closed habitat during the Pleistocene-Holocene transition (Supplementary Fig. S2). However, this study does not permit to make inferences on the timing of an ecological shift among hunter-gatherers in the area based on the proposal of one human isotope sample, which is likely to be biased. Overall, our carbon isotope data advocate that highland Pang Mapha provided an unprecedentedly optimal niche with broad-spectrum resources for the survival of hunter-gatherers during the terminal Pleistocene.

### Hunter-gatherer mobility and adaptation during the late Pleistocene

Along the major routes of human migration from Africa to Australia and across SEA, recent studies have shown that tropical rainforests, previously thought as an unfavorable niche for the early human occupation^71^, have successfully been exploited by some hunter-gatherers for at least 45 ka (e.g., Africa^72–74^, Sri Lanka^75–78^, South China^12,79^, Vietnam^80–82^, Borneo^83–85^, Sumatra^86,87^, Java^88,89^, Timor^90^, Papua^91^, and New Guinea^92–94^). Despite the seemingly specialized occupation of coastal resources by other human populations in ISEA within Wallacea, stable isotope compositions of human and faunal tooth enamel in Alor suggested the possible availability of C_4_ resources used by late Pleistocene small mammals and hunter-gatherer populations (40–21 ka)^90^. All the evidence has suggested so far that more specialized hunting of arboreal and semi-arboreal mammals and the occupation of tropical rainforest habitats have commonly been favored among the late Pleistocene hunter-gatherer populations from these aforementioned regions. Unlike the long-established rainforest occupation by those late Pleistocene hunter-gatherers, our isotopic data provide direct evidence for human reliance on the different types of habitats (dated around 19–12 ka) in MSEA.

Based on available zooarchaeological information on the taxonomic diversity, species abundance, and mortality profiles of successive faunal assemblages from TLR, it has been suggested that hunter-gatherers in the area adopted a generalized and mixed subsistence strategy^30,31^. Studies of lithic assemblages have shown that classical sumatralith forms such as flakes and radial cores made from metaquartzites or sandstones were found from TLR, while microliths indicative of specialized rainforest hunting by *Homo sapiens*, as discovered in the archaeological sites of Sri Lanka about ~ 45,000 years ago^78,95^, were absent in the area^26,27,29^. The analysis of ecological models applied to flaked stone artefacts in TLR have suggested that proximity to resources and climate change were important factors influencing Hoabinhian technology^26,29^. In addition, the stable isotope data obtained in the present study allow us to evaluate directly the degree of early modern human reliance on a broad spectrum of resources and to refine the relationship between the lithic assemblages and prevailing open environments in TLR. Environmental heterogeneity in highland Pang Mapha allowed *Homo sapiens* to have more opportunistic foraging on various types of preys and more available C_3_- and C_4_-based resources to exploit during the terminal Pleistocene. The population of late Pleistocene hunter-gatherers in Pang Mapha might have selected a settlement option at the edge of open forests where they could take advantage to exploit both closed and open canopy resources, not a particular area in one extreme direction of environmental ranges. Compared to the timing of microlithic records in South Asia, our study reinforces the late occurrence of the proliferation of microliths in highland MSEA. The high environmental heterogeneity resulted in low ecological stress in forcing and shaping behavioural adaptations among the late Pleistocene hunter-gatherers, allowing the persistence of similar subsistence patterns and stone artefact technology in the area for at least 20,000 years.

As it is visible in the settlement of highland Northwest Thailand by early modern humans during the late Pleistocene, they have ventured into high-altitude mountains where the savanna ecosystem was present. Although the TLR hunter-gatherers had the mixed grassland and woodland adaptations comparable to other earlier hominins such as *Homo erectus* and *Homo florensiensis* in ISEA (e.g.,^96,97^), the degree and direction of ecological reliance between *Homo sapiens* and other hominin species on the broad spectrum of resources and environments might be questioned and require future studies. However, our findings stand in striking contrast to the specialized rainforest occupation among other contemporaneous hunter-gatherers in MSEA’s neighboring continents/regions. Our isotopic evidence not only suggests the asynchronous occurrence of specialized rainforest occupations among the hunter-gatherer populations in MSEA, but also documents diverse late Pleistocene human adaptations during the process of dispersal towards Wallacea and Sahul.

## Methods

### Sample collection and design

We analyzed a total of 139 bulk tooth enamel samples of humans and non-human mammals from the late Pleistocene archaeological sites of Tham Lod Rockshelter in highland Pang Mapha. Five high-crowned teeth of large bovids from TLR (45 samples) were selected for serial sampling. All teeth were collected along each well-dated layer from one stratigraphic side of the wall (west profile) in the Area 1 of TLR, in order to ensure the vertical placement of samples (Fig. 2). Sixteen samples of dentine and five samples of soil carbonates were additionally analyzed for isotopic comparisons (see Supplementary Information 2, Supplementary Table S8, and Supplementary Fig. S4 for isotope datasets and interpretations). All available dates and geological information for the site are given in Supplementary Information 1 and Supplementary Table S1).

### Stable carbon and oxygen isotope analysis of carbonates

For preliminary measurements in the study, we performed a CNS elemental analysis to test the possibility of collagen preservation in bone and dentine samples from Tham Lod Rockshelter (Supplementary Table S3). The CNS analysis was conducted at the Laboratory for Soil Science and Geoecology (University of Tübingen) using a Vario EL III elemental analyzer. As is the case for the site studied here, the bone and dentine collagen has highly been degraded over most of the stratigraphic layers and is nearly impossible to be extracted. Therefore, the isotopic data were retrieved from the bioapatite carbonate of tooth enamel.

Bulk enamel powder was sampled along the whole length of a tooth crown in order to obtain average isotopic signals over the time period of dental growth, while serial sampling was taken along a non-occlusal surface parallel to the growth axis and across its entire length (lengthwise from the top to the bottom of tooth crowns) on the labial side of lower molars. The isotopic pretreatment and measurements of samples were performed at the Department of Geosciences, University of Tübingen, Germany (see Supplementary Information 3 for more detailed information on the isotopic pretreatment and protocols). Stable carbon and oxygen isotopic values are expressed as the following standard delta (*δ*)–notation: X = [(R_sample_/R_standard_) − 1]*1000, where X stands for ^13^C and ^18^O values and R is referred to ^13^C/^12^C or ^18^O/^16^O, respectively. The recorded delta values follow the international reference standards, “Vienna PeeDee Belemnite” (VPDB) for the carbon and oxygen. *δ*^18^O values relative to “Vienna Standard Mean Ocean Water” (VSMOW) are also given. The carbonate content (CaCO_3_%) was calculated using the ratio between amount of CO_2_ released by the reaction, as detected from the peak intensity for mass 44 and the weight of pure carbonate used as a standard, with an analytical error of 0.3%, based on the multiple analysis of reference enamel samples.

### Statistical analysis

Using the Shapiro–Wilk test, our samples with unequal variances do not correspond to a normal distribution. In the case for which at least five samples are available, *δ*^13^C and *δ*^18^O values were statistically tested for examining a significant difference among our analyzed enamel dataset. We thus performed non-parametric tests to analyze differences in median *δ*^13^C and *δ*^18^O values among our isotopic samples within the locality (Kruskal–Wallis test) and between the species (Mann–Whitney U-test) (Supplementary Table S5). Significant differences are statistically attained when p-values are equal to or less than 0.05. All statistical analyses were carried out using the software PAST version 4^98^.

## Supplementary Information

Supplementary Information 2.

Supplementary Information 1.
