# Supplementary material for: Late Pleistocene human paleoecology in the highland savanna ecosystem of mainland Southeast Asia
Source: Sci Rep. 2021 Aug 18;11:16756. doi: 10.1038/s41598-021-96260-4 (PMC8373907; doi:10.1038/s41598-021-96260-4)
Supplement: Supplementary file 1 — Supplementary Information 2. [file 41598_2021_96260_MOESM1_ESM.pdf]

Supplementary Information for

Late Pleistocene human paleoecology in the highland savanna ecosystem of mainland Southeast Asia

Kantapon Suraprasit, Rasmi Shoocongdej, Kanoknart Chintakanon, and Hervé Bocherens

This PDF file includes:

- Supplementary Information 1 to 3
- Tables S1 to S8
- Figures S1 to S4
- SI References

Supplementary Information 1. General information, geological backgrounds, and available dates of the study area.

Geographic location and modern climate and ecosystems of Pang Mapha

Highland Pang Mapha covering 1,210 km^2 is a district of Mae Hong Son province, northwestern Thailand. It is located at the border between Thailand and the Shan state of Myanmar. This area yields a plenty of a karst topography bearing the north-south limestone mountain ranges, with the average elevation ranging from 600 to 1,170 m above mean sea level. There are five river drainages including Mae Lana, Pong Sean Pik, Khlong, Lang, and Rang Luang in the region. The landscape of the area is characterized by the majority of high mountains (90%) with some parts of valleys (10%) consisting of the high and low alluvium ([1]). Several caves and rockshelters have been formed in limestone mountains of Pang Mapha. This area is today under the tropical monsoon climate, with well-pronounced wet (May to October) and dry (November to February) seasons, and experiences the average annual rainfall of approximately 1300 mm. The vegetation cover is classified into 6 types including mixed deciduous, dry dipterocarp, hill evergreen, dry evergreen, bamboo, and limestone forests ([2]; [3]).

Tham Lod Rockshelter

The archaeological site of Tham Lod Rockshelter (19°34′ N, 98°16′ E, about 150 km northwest of Chiang Mai), with an elevation of approximately 640 m above sea level, is located close to the Lang River at the Pang Mapha District, Mae Hong Son Province in northwestern Thailand. Three trenches (so called "Area 1", "Area 2", and "Area 3") positioned at the shelter floor along the cliff of a Permian limestone karst were excavated by the Highland Archaeology Project in 2002. The archaeological material has been analyzed for three years (between 2003 and 2006). The first archaeological trench or the Area 1, with the dimension of 6 m^2 and the depth of about 5 m, is focused here for this study and was dug closest to the wall of a rock shelter where a prehistoric hunter-gatherer occupation was located. The excavation sequence of the Area 1 (west profile) is divided into three main units (unit A, B, and C, from the surface to the bottom) based on the

archaeological culture distinctions ([4]) and into ten stratigraphic units, from 1 (surface) to 10 (the deepest excavation level), on the basis of the sedimentological analysis ([5]). In the geological context, the homogeneous unconsolidated soils deposited in the Area 1 (from the unit 1 to 8) are somewhat mixed by the human nature and cultural processes. A discontinuity in the deposition of soil layers is observed along the vertical stratigraphic section of the archaeological excavation. Mammal fossils were often found from the top soil to the unit 7 (about 3.5 m deep from the surface) but were very rare in the unit 8. Other two deeper units 9 and 10 correspond to gravel and sterile deposits, respectively, where none of animal remains have been found ([5]). According to the systematically archaeological excavations of the Area 1 where every interval of 10-20 cm depth of an unconsolidated soil deposit was dug up and screened for each fossil detected, the stratigraphic profile of the west wall can be divided into 45 layers (4.5 m deep).

Mammal assemblages from the deposits of Tham Lod Rockshelter consisted of a highly diverse groups of modern taxa that are living in Thailand, but some locally extinct species such as *Rhinoceros sondaicus* and *N. goral* were also present ([6]). Two burials of human skeletal remains (adult male and female) and other two fragmentary individuals as well as stone tools, flakes, and potsherds were found from the upper part of the Area 1, where direct dating of organic sediments adjacent to each of the adult individuals provided ages of 14,070±140 yr BP (male) and 14,764±60 yr BP (female) ([4]; [7]). Regarding the stratigraphic sequence of the Area 1, dating analyses using radiocarbon (^{14}C) and thermoluminescence (TL) methods performed on organic material and sediments provided an age ranging from 32,380±292 yr BP (Akita-TL10) to 12,100±60 yr BP (Beta-168223) for the geological unit 6 to unit 3 ([4]; [5]; [7]; [8]). Although the AMS radiocarbon date for charcoal has shown an inverse age for the layer 17 of the Area 1, [7] suggested that the radiocarbon age of this layer is unlikely or unnatural because the dated sample might have originated from roof fall events. However, rockshelter settings commonly show evidence of disturbed or mixed contexts throughout their formation, which is caused by frequent and intermittent human agency. Resolving this issue, [9] conducted radiocarbon datings on the fresh water bivalve *Margaritanopsis laosensis* recollected along every interval of 10-20 cm depth throughout the stratigraphic sequence of the

Area 1 (west profile). These radiocarbon dates on shells have provided a continuous series of ages ranging from 34,130 yr BP to 11,180 yr BP for the layer 31 to layer 1 (see Supplementary Table S1 for detailed dates).

Although there was a possibility of disturbances by modern activities that might have brought more recent samples into sediment layers near the ground surface (i.e. layer 1, 2, and 3) ([7]), it is likely that the stratigraphic sequence of Tham Lod Rockshelter shows no evidence of major disturbances and appears to represent an increasing accumulation of sediments over time, with a high degree of stratigraphic integrity ([4]; [7]; [9]; [10]).

Supplementary Information 2. Dentine and soil carbonate isotope data and interpretations.

Dentine samples analyzed within the same individuals of mammals often showed lower $\delta^{13}C$ values compared to tooth enamel for those of grazers but higher $\delta^{13}C$ values than or rather similar $\delta^{13}C$ values for those of browsers (Supplementary Table S4 and Supplementary Fig. S4). Differences in $\delta^{13}C$ between dentine and enamel probably reflected the dietary resources used by these mammals during the different timing/developmental stages ([11]). Samples of soil carbonates collected from different stratigraphic layers of TLR yielded a median of -15.3‰ (range: -20.3‰ to -13.2‰, n=5) for $\delta^{13}C$ values and a median of -10.2‰ (range:-13.7‰ to -8.1‰, n=5) for $\delta^{18}O$ (Supplementary Table S8). The lower $\delta^{13}C$ values of soil carbonates than those of enamel suggested potential isotopic alteration caused by diagenesis.

Supplementary Information 3. Stable isotope protocols of tooth enamel, dentine, and soil carbonate: sampling process and pretreatments.

Before sampling, we removed the outermost surface of tooth enamel and dentine. Enamel and dentine powder and soil carbonate (10-15 mg) were collected using a diamond-tipped drill with a high-speed rotary tool. Sample powder collected in 1.5 ml vials was treated by adding a solution of 1.35 ml 25% NaOCl. The solution was mixed by a vortex, shaken for 24 hours and subsequently rinsed three times with Milli-Q H_2O in order to remove organic material. After rinsing, soil carbonate was dried in an oven at 35 °C for 72 hours but 1.35 ml of 1 M Acetic Acid Buffer solution was added to the vials of enamel and dentine samples and then shaken again for 24 hours in order to remove secondary carbonates ([11]; [12]; [13]). The solution was rinsed another three times with Milli-Q H_2O to remove the buffer solution and dried in an oven at 35 °C for 72 hours to obtain the structural carbonate ([13]).

Pretreated (enamel, dentine, and soil) carbonate was reacted with 103% H_3PO_4 for 4 hours at 70 °C using a MultiFlow-Geo interfaced with the Elementar IsoPrime 100 IRMS. Final isotopic ratios are reported per mil (‰) calibrated with international standards (IAEA-603: $\delta^{13}C$= 2.46‰/$\delta^{18}O$= -2.37‰ and NBS-18: $\delta^{13}C$= -5.014‰/$\delta^{18}O$= -23.2‰), as well as three in-house standards. IonOS software (Version 3.2) by Elementar was used to carry out multi-point standard isotope calibration by generating a trend line (y=mx+c) that maps measured versus expected isotopic results, which is then used to calibrate sample results. The measurement uncertainty was monitored using three in-house standards. The overall analytical precision is higher than 0.1‰ for carbon and better than 0.2‰ for oxygen isotopic values.

Supplementary Table S1. Radiocarbon (^{14}C) and thermoluminescence (TL) dates for the Area 1 of Tham Lod Rockshelter (data from [4] and [9]). All calibrated radiocarbon ages are quoted in year before present (BP), where present is 1950 AD.

Tham Lod Rockshelter, Area 1					
Layer	Arbitrary depth (cm.dt)	Depth from surface (cm)	^{14}C dates on bivalves (yr BP)	^{14}C dates on other material (yr BP±1σ)	TL dates on quartz and calcrete (yr BP±1σ)
S	S-167	3-4			
1	150-168.5	5-7	11,180		
2	155-170	5-20			
3	170-180	30-40			
4	180-190	40-50	12,770		
5	190-200	50-60	13,560	14,070±140 (Beta-168223)	
6	200-210	60-70	14,350		
7	210-220	70-80	15,140	14,764±60 (Beta-168224)	
8	220-230	80-90	15,930		
9	230-240	90-100			13,422±541 (Akita-TL7)
10	240-250	100-110	17,510		
11	250-260	110-120	18,310		
12	260-270	120-130	19,100		
13	270-280	130-140	19,890		
14	280-290	140-150	20,680		
15	290-300	150-160			
16	300-310	160-170	22,260		
17	310-320	170-180	23,050	29,910±270 (Beta-194122)	
18	320-330	180-190	23,840		
19	330-340	190-200	24,640		
20	340-350	200-210	25,430		
21	350-360	210-220	26,220		
22	360-370	220-230	27,010		
23	370-380	230-240	27,800		
24	380-390	240-250	28,590		22,257±154 (Akita-TL12)
25	390-400	250-260	29,380		

26	400-410	260-270	30,180		
27	410-420	270-280	30,970		
28	420-430	280-290	31,760	26,740±400 (Beta-172226)	
29	430-440	290-300	32,550		
30	440-450	300-310	33,340		
31	450-460	310-320	34,130		32,380±292 (Akita-TL10)
32	460-470	320-330			
33	470-480	330-340			
34	480-490	340-350			
35	490-500	350-360			

Supplementary Table S2. List and NISP of mammalian faunas from Tham Lod Rockshelter in highland Pang Mapha (modified from [6]).

	**Tham Lod Rockshelter**			
**Excavation area**	**Area 1**	**Area 2**		
**Trench**	**S23W10**	**S21W10**	**Baulk S21W10**	**S20W10**
PRIMATES				
Macaca sp.	13			
Cercopithecidae indet.	17			
Colobinae indet.	4			
RODENTIA				
Rhizomys sp.	2			
Cannomys badius	10			
Rhizomyinae indet.	28	7		
Bandicota indica	1			
Bandicota sp.	1			
Hystrix sp.	34	4		
CARNIVORA				
Ursus thibetanus/ Ursidae indet.	44	6		1
Arctonyx collaris	1			
Panthera tigris	3			
Carnivora indet.	36			
PROBOSCIDEA				
Elephas sp.	6			
PERISSODACTYLA				
Rhinoceros sondaicus	1	1	4	
Rhinocerotidae indet.	3			
ARTIODACTYLA				
Sus scrofa	90	8	2	
Muntiacus sp.	37	4		2
Axis porcinus	233	55	4	1
Rucervus eldii	11	49	11	
Rusa unicolor	74	327	14	4

Rucervus sp./*Rusa* sp.	376	7		
Cervidae indet.	155	196	23	6
Bos gaurus	43	28	10	5
Bos javanicus	16	19	1	5
Bos sp.	4			
Bubalus arnee	8	12		
Naemorhedus goral	16	3		1
Naemorhedus griseus	111	23	6	7
Capricornis sumatraensis	31	4		1
Total	1,409	753	75	33

Supplementary Table S3. Nitrogen and carbon concentrations (wt. %N and wt. %C) in dentine and bone samples from the Area 1 of Tham Lod Rockshelter. To test the possibility of collagen preservation, the examination of the percent nitrogen of bone and dentine powder follows the protocol of [14]. The usable samples or collagen dentine and bones should have the nitrogen content higher than 0·5 wt% but should be also checked for further indications of breakdown of sample integrity ([15]; [16]).

**Specimen no.**	**Element**	**Taxon**	**Layer**	**N%**	**CORR C%**
A5	Dental root	*Rusa unicolor*	1	0.04	3.18
A11	Dentine	Rhinocerotid indet.	1	0.02	0.00
A19	Mandibular bone	*Rusa unicolor*	2	0.07	3.32
A118	Dental root	*Rusa unicolor*	2	0.04	2.08
A4278	Mandibular bone	*Sus scrofa*	2	0.05	4.75
A49	Dental root	*Bos* sp.	3	0.09	1.93
A263	Mandibular bone	*Naemorhedus griseus*	5	0.05	11.64
A277	Dentine	*Rusa unicolor*	5	0.09	2.47
A516	Mandibular bone	*Ursus thibetanus*	6	0.04	6.30
A738	Dentine	*Capricornis sumatraensis*	7	0.06	1.67
A910	Mandibular bone	*Rucervus eldii*	7	0.07	1.62
A1182	Dental root	*Ursus thibetanus*	10	0.05	2.83
A1292	Dental root	*Bos gaurus*	11	0.05	3.59
A1542	Mandibular bone	*Naemorhedus griseus*	12	0.05	3.01
A1621	Mandibular bone	*Rusa unicolor*	14	0.04	2.50
A2210	Dental root	*Bubalus arnee*	17	0.17	3.01
A3820	Mandibular bone	*Rucervus eldii*	21	0.04	4.65
A7844	Mandibular bone	*Rusa unicolor*	29	0.06	2.10
A7879	Mandibular bone	*Rusa unicolor*	29	0.04	3.38

Supplementary Table S4. Bulk δ^{13}C and δ^{18}O values and calcium carbonate content of human and faunal tooth enamel and dentine from the Area 1 of Tham Lod Rockshelter in Pang Mapha, northwestern Thailand.

**Specimen no.**	**Taxa**	**Teeth**	**Tissues**	**$CaCO_3$ (%)**	**δ^{13}C (‰VPDB)**	**δ^{18}O (‰VPDB)**	**δ^{18}O (‰VSMOW)**	**Layers**	**Remarks**
**Tham Lod Rockshelter**									
A4293	*Macaca* sp.	m1	Enamel	3.7	-15.2	-0.6	30.3	4	
A116	*Macaca* sp.	m2	Enamel	5.6	-1.9	-5.6	25.1	4	
			Dentine	8.0	-5.5	-5.2	25.5	4	
A260	*Macaca* sp.	m2	Enamel	3.4	-16.0	-4.0	26.7	5	
A5350	*Macaca* sp.	m3	Enamel	4.5	-14.2	-6.9	23.7	23	
A6486	*Macaca* sp.	p4	Enamel	4.3	-16.0	-7.8	22.9	24	
A6209	*Macaca* sp.	m2	Enamel	3.6	-14.8	-7.3	23.4	24	
A6487	*Macaca* sp.	m2	Enamel	3.3	-15.1	-8.6	22.0	24	
A7673	*Macaca* sp.	m1	Enamel	4.0	-16.0	-4.4	26.3	27	
611	*Homo sapiens*	P3	Enamel	3.9	-14.0	-7.9	22.7	3	Burnt material
607	*Homo sapiens*	c1	Enamel	2.4	-9.6	-6.7	23.9	4	
			Dentine	3.3	-9.4	-7.3	23.3	4	
620	*Homo sapiens*	M3	Enamel	2.1	-10.2	-7.0	23.6	4	
618	*Homo sapiens*	M3	Enamel	3.2	-9.4	-6.7	24.0	5	
652	*Homo sapiens*	dm2	Enamel	4.0	-10.3	-5.4	25.3	12	
A42	*Hystrix* sp.	I1/i1 fragment	Enamel	2.7	-12.6	-7.8	22.9	3	
A62	*Hystrix* sp.	I1/i1 fragment	Enamel	3.5	-13.4	-4.9	25.8	3	
A841	*Hystrix* sp.	I1/i1 fragment	Enamel	2.6	-12.7	-6.4	24.2	7	
A911	*Hystrix* sp.	I1 fragment	Enamel	2.0	-13.4	-5.1	25.6	7	
A1697	*Hystrix* sp.	I1/i1 fragment	Enamel	2.6	-12.2	-6.6	24.1	14	
A2571	*Hystrix* sp.	P4	Enamel	4.3	-14.6	-7.9	22.7	18	
A3011	*Hystrix* sp.	P4	Enamel	4.8	-11.4	-6.8	23.8	19	
A5187	*Hystrix* sp.	I1/i1 fragment	Dentine	4.8	-10.3	-6.9	23.7	22	
A5805	*Hystrix* sp.	p4	Enamel	4.0	-15.3	-8.5	22.1	23	
A7397	*Hystrix* sp.	M fragment	Enamel	4.9	-13.3	-7.9	22.7	26	
A7448	*Hystrix* sp.	M fragment	Enamel	4.6	-12.9	-7.5	23.2	27	
A7449	*Hystrix* sp.	M1 or M2	Enamel	4.5	-14.0	-5.5	25.2	27	
A517	Rodentia indet.	i1 fragment	Enamel	3.2	-9.5	-6.3	24.4	6	
A1667	Rodentia indet.	I1 fragment	Enamel	3.4	-12.5	-7.1	23.5	14	
A1699	Rodentia indet.	I1/i1 fragment	Enamel	3.1	-13.2	-7.6	23.0	14	
A2374	Rodentia indet.	I1/i1 fragment	Enamel	3.4	-13.8	-6.1	24.6	17	
A2375	Rodentia indet.	I1 fragment	Enamel	3.3	-13.3	-6.6	24.1	17	

A3534	Rodentia indet.	I1 fragment	Enamel	2.9	-6.7	-7.8	22.8	20	
A3887	Rodentia indet.	i1 fragment	Enamel	3.4	-0.4	-7.9	22.7	21	
A7858	Rodentia indet.	I1/i1 fragment	Enamel	3.3	-7.8	-7.1	23.6	29	
			Dentine	5.3	-9.0	-6.9	23.8	29	
A4288	*Ursus thibetanus*	i3	Enamel	5.0	-13.7	-5.5	25.2	4	
A25	*Ursus thibetanus*	M1 fragment	Enamel	4.3	-15.3	-5.8	24.9	4	Data from [17]
A105	*Ursus thibetanus*	M2	Enamel	5.0	-13.3	-6.7	23.9	4	
A108	*Ursus thibetanus*	m1 fragment	Enamel	4.2	-14.4	-5.0	25.7	4	
A109	*Ursus thibetanus*	p4	Enamel	4.9	-13.2	-5.9	24.7	4	
A134	*Ursus thibetanus*	C1	Enamel	4.4	-12.6	-5.4	25.3	4	
			Dentine	6.3	-10.6	-6.5	24.1	4	
A268	*Ursus thibetanus*	P4	Enamel	4.7	-13.5	-5.2	25.5	5	
A498	*Ursus thibetanus*	M1	Enamel	4.0	-13.8	-5.5	25.2	6	Data from [17]
A516	*Ursus thibetanus*	M2	Enamel	3.8	-13.8	-5.7	24.9	6	
A541	*Ursus thibetanus*	i1	Enamel	3.8	-13.5	-5.6	25.1	6	
A1182	*Ursus thibetanus*	I3	Enamel	5.7	-3.5	-6.1	24.6	10	
A1464	*Ursus thibetanus*	C1	Enamel	4.3	-12.8	-6.1	24.5	12	
			Dentine	5.7	-11.5	-6.5	24.2	12	
A3228	*Ursus thibetanus*	P4	Enamel	3.3	-11.8	-2.4	28.3	20	
			Dentine	5.8	-11.5	-7.2	23.4	20	
A3229	*Ursus thibetanus*	P4	Enamel	5.0	-13.2	-5.1	25.6	20	
A5028	*Ursus thibetanus*	M2 fragment	Enamel	4.9	-13.7	-7.2	23.4	22	
A5029	*Ursus thibetanus*	P4	Enamel	4.7	-14.1	-6.3	24.3	22	
A5174	*Ursus thibetanus*	M1	Enamel	4.1	-13.9	-6.5	24.1	22	
A5233	*Ursus thibetanus*	C1	Enamel	4.6	-12.3	-7.0	23.7	23	
A5234	*Ursus thibetanus*	M2 fragment	Enamel	4.6	-13.1	-6.2	24.5	23	Data from [17]
A5707	*Ursus thibetanus*	P4	Enamel	3.9	-14.6	-7.6	23.0	23	Burnt material
A5708	*Ursus thibetanus*	C1	Enamel	4.4	-13.6	-7.1	23.5	23	
A5806	*Ursus thibetanus*	p1	Enamel	4.1	-14.3	-6.3	24.4	23	
A6208	*Ursus thibetanus*	M1 fragment	Enamel	4.2	-14.4	-9.5	21.1	24	
A6851	*Ursus thibetanus*	M2 fragment	Enamel	3.4	-13.4	-9.2	21.4	25	
A7698	*Ursus thibetanus*	C1 fragment	Enamel	4.3	-11.9	-6.3	24.4	28	
A6751	*Arctonyx collaris*	m1	Enamel	5.1	-10.9	-8.0	22.6	25	
A4278	*Sus scrofa*	M/m fragment	Enamel	6.2	-4.3	-5.1	25.7	2	
			Dentine	6.7	-9.9	-6.8	23.8	2	
A136	*Sus scrofa*	M/m fragment	Enamel	4.9	-10.3	-10.9	19.7	4	
A730	*Sus scrofa*	m1 fragment	Enamel	4.7	-10.9	-7.0	23.6	7	
A1469	*Sus scrofa*	m3	Enamel	3.1	-3.1	-8.7	21.8	12	

A2005	*Sus scrofa*	m3 fragment	Enamel	2.8	-6.2	-8.7	21.9	16	
A2486	*Sus scrofa*	m3 fragment	Enamel	3.0	-10.1	-9.9	20.7	18	
A2788	*Sus scrofa*	m1 fragment	Enamel	5.0	-11.1	-8.1	22.5	19	
			Dentine	5.8	-10.9	-7.4	23.3	19	
A2791	*Sus scrofa*	m2	Enamel	4.6	-9.8	-7.8	22.8	18.5	
A3226	*Sus scrofa*	m3 fragment	Enamel	3.0	-7.9	-9.7	20.8	20	Data from [17]
A5150	*Sus scrofa*	m3 fragment	Enamel	2.8	-6.9	-10.1	20.4	22	Data from [17]
A5384	*Sus scrofa*	m3 fragment	Enamel	2.8	-4.2	-9.1	21.5	23	
A6190	*Sus scrofa*	M/m fragment	Enamel	3.0	-9.3	-11.2	19.3	24	
A174	*Sus scrofa*	m3 fragment	Enamel	2.6	-3.9	-10.1	20.4	24.5	
A7839	*Sus scrofa*	P3	Enamel	4.7	-7.3	-8.8	21.8	29	
A41	*Panthera tigris*	I3	Enamel	5.0	-11.5	-7.0	23.6	3	
A3416	*Panthera tigris*	C1	Enamel	4.6	-6.0	-7.2	23.5	20	
A5697	*Panthera tigris*	I3	Enamel	3.6	-13.0	-7.4	23.3	23	Data from [17]
A1491	*Elephas* sp.	M/m fragment	Enamel	5.5	-4.7	-8.2	22.4	12	
A11	Rhinocerotidae indet.	m fragment	Enamel	6.5	1.0	-5.3	25.4	1	
			Dentine	6.1	1.2	-5.2	25.6	1	
A731	Rhinocerotidae indet.	m fragment	Enamel	4.7	-14.6	-5.3	25.4	7	
A4990	Rhinocerotidae indet.	m fragment	Enamel	3.8	-0.1	-6.6	24.1	22	
			Dentine	3.8	-2.4	-9.1	21.5	22	
A3	*Muntiacus* sp.	DP3	Enamel	5.0	-13.7	-6.6	24.1	1	
A1915b	*Muntiacus* sp.	P4	Enamel	3.8	-12.3	-8.5	22.1	17	
A7912	*Muntiacus* sp.	M1	Enamel	3.0	-12.6	-5.0	25.7	31	
A24	*Axis porcinus*	P4	Enamel	3.5	-13.8	-7.9	22.7	2	
A1028	*Axis porcinus*	m1	Enamel	4.7	-14.7	-2.7	28.0	9	
A3817	*Axis porcinus*	m2	Enamel	3.8	-12.6	-9.7	20.8	21	
A120	*Rucervus eldii*	m3	Enamel	3.8	2.7	-4.0	26.7	4	
A121	*Rucervus eldii*	M3	Enamel	4.8	3.1	-7.3	23.4	4	
A910	*Rucervus eldii*	m3	Enamel	3.3	2.3	-5.5	25.1	7	Data from [17]
A1412	*Rucervus eldii*	m3	Enamel	3.3	3.2	-7.7	23.0	12	
A1512	*Rucervus eldii*	m2	Enamel	3.7	2.7	-4.5	26.2	13	
A1681b	*Rucervus eldii*	p3	Enamel	3.9	1.5	-6.1	24.6	14	Data from [17]
A1746	*Rucervus eldii*	m3	Enamel	4.2	2.3	-4.1	26.7	15	
A2483	*Rucervus eldii*	m3	Enamel	3.2	3.2	-6.6	24.1	18	
A3666	*Rucervus eldii*	m3	Enamel	3.5	3.4	-8.3	22.3	20	
A3820	*Rucervus eldii*	m3	Enamel	3.3	2.4	-5.7	24.9	21	
A5041	*Rucervus eldii*	m3	Enamel	5.6	1.7	-5.0	25.7	22	
A5	*Rusa unicolor*	M2	Enamel	5.5	-6.2	-2.9	27.9	1	

A19	*Rusa unicolor*	m2	Enamel	2.7	-2.2	-6.2	24.4	2	
A23	*Rusa unicolor*	M3	Enamel	3.3	-1.3	-5.4	25.3	2	
A48	*Rusa unicolor*	M2 fragment	Enamel	2.6	-2.9	-6.8	23.9	3	
A106	*Rusa unicolor*	m3	Enamel	3.5	-4.9	-4.8	25.9	4	
A124	*Rusa unicolor*	m fragment	Enamel	2.8	-4.4	-6.5	24.1	4	
A125	*Rusa unicolor*	m1 or m2	Enamel	6.0	0.5	-5.8	24.9	4	
A126	*Rusa unicolor*	P4	Enamel	3.2	-4.5	-3.8	26.9	4	
A137	*Rusa unicolor*	M3	Enamel	2.9	-4.0	-5.4	25.3	4	
A277	*Rusa unicolor*	m1	Enamel	5.0	-2.7	-4.7	26.0	5	
			Dentine	5.0	-7.3	-6.6	24.0	5	
A278	*Rusa unicolor*	m2	Enamel	3.1	-5.2	-8.6	22.0	5	Data from [17]
A514	*Rusa unicolor*	M2	Enamel	3.4	-1.6	-7.1	23.5	6	
A537	*Rusa unicolor*	m3	Enamel	3.1	-1.4	-8.2	22.4	6	
A830	*Rusa unicolor*	m3	Enamel	3.2	0.1	-6.8	23.8	7	
A1617	*Rusa unicolor*	P3	Enamel	3.2	-1.3	-5.7	25.0	14	
A1620	*Rusa unicolor*	m3	Enamel	3.1	-3.1	-7.0	23.6	14	
A1621	*Rusa unicolor*	m3	Enamel	2.9	-1.8	-5.8	24.9	14	
A2948	*Rusa unicolor*	m3	Enamel	3.1	-4.3	-9.2	21.4	19	
A3436	*Rusa unicolor*	m1	Enamel	3.3	0.2	-9.1	21.5	20	
A5179	*Rusa unicolor*	m3	Enamel	2.8	-3.8	-8.7	21.9	22	
A6199	*Rusa unicolor*	m2	Enamel	4.9	-3.4	-3.6	27.1	24	
A6200	*Rusa unicolor*	m3	Enamel	3.1	-2.6	-6.9	23.8	24	
A7089b	*Rusa unicolor*	m1	Enamel	4.2	-5.6	-5.0	25.7	26	
A7539	*Rusa unicolor*	m1	Enamel	3.9	-0.2	-7.9	22.7	27	
A7695	*Rusa unicolor*	m2	Enamel	3.3	-4.9	-8.0	22.6	28	Data from [17]
A7721	*Rusa unicolor*	M2	Enamel	3.1	-0.2	-7.5	23.1	28	
A7835	*Rusa unicolor*	M3	Enamel	3.7	-3.2	-10.3	20.2	29	
A7838	*Rusa unicolor*	m fragment	Enamel	2.8	-1.3	-8.6	22.0	29	
			Dentine	7.1	-6.0	-6.7	23.9	29	
A7844	*Rusa unicolor*	p4	Enamel	3.1	-0.8	-6.6	24.0	29	
A7879	*Rusa unicolor*	m1	Enamel	3.8	-4.2	-7.7	23.0	29	
A7894	*Rusa unicolor*	P4	Enamel	4.8	-1.6	-4.4	26.3	30	
A7907	*Rusa unicolor*	m fragment	Enamel	2.8	-0.9	-8.2	22.4	30.5	
A7909	*Rusa unicolor*	P	Enamel	5.5	2.0	-7.4	23.2	31	
A7910	*Rusa unicolor*	M1	Enamel	3.5	-2.1	-10.8	19.8	31	
A7911	*Rusa unicolor*	M fragment	Enamel	6.4	-3.6	-8.5	22.1	31	
A1538	*Bubalus arnee*	P3	Enamel	4.2	0.3	-5.2	25.5	13	
A2210	*Bubalus arnee*	P2	Enamel	4.2	2.4	-6.4	24.2	17	Data from [17]
A5236	*Bubalus arnee*	p3	Enamel	3.8	3.0	-7.5	23.1	23	Data from [17]
A6742	*Bubalus arnee*	M3	Enamel	3.7	4.6	-5.5	25.2	25	

A6743+A6845	*Bubalus arnee*	M1 or M2	Enamel	3.4	4.7	-6.3	24.4	25	
A770	*Bos javanicus*	P4	Enamel	3.9	2.8	-6.2	24.5	7	
A915	*Bos javanicus*	m1	Enamel	3.6	2.4	-6.4	24.2	7	Average value of serial sampling
A920	*Bos javanicus*	m2	Enamel	4.2	1.5	-5.3	25.4	7	
A131	*Bos gaurus*	p4	Enamel	3.9	3.4	-3.5	27.3	4	
A927	*Bos gaurus*	m2	Enamel	4.1	1.3	-6.2	24.4	8	
A934	*Bos gaurus*	m2	Enamel	3.5	1.8	-4.0	26.7	8	
A1166	*Bos gaurus*	M1	Enamel	4.4	-0.3	-7.0	23.6	10	
A1207	*Bos gaurus*	m2	Enamel	4.2	-0.3	-8.1	22.6	10	Average value of serial sampling
A1292	*Bos gaurus*	m3	Enamel	4.4	1.0	-6.5	24.2	11	
A1606	*Bos gaurus*	m2	Enamel	6.0	-0.9	-5.3	25.4	14	
A1677	*Bos gaurus*	m1	Enamel	4.6	0.9	-5.2	25.5	14	Average value of serial sampling
A1720b	*Bos gaurus*	m2	Enamel	4.2	1.2	-6.0	24.7	14	
A1791	*Bos gaurus*	m1	Enamel	4.2	0.4	-6.1	24.6	15	
			Dentine	4.8	-2.4	-5.5	25.2	15	
A3697	*Bos gaurus*	m2	Enamel	3.6	0.0	-8.0	22.7	21	Average value of serial sampling
A4210	*Bos gaurus*	m2	Enamel	3.5	1.2	-5.1	25.6	21	
A7863b	*Bos gaurus*	m2	Enamel	2.3	3.3	-8.8	21.8	29	
A49	*Bos* sp.	m1	Enamel	3.5	2.1	-6.5	24.2	3	
A275	*Bos* sp.	M/m fragment	Enamel	5.7	2.1	-5.5	25.2	5	
A1291	*Bos* sp.	m fragment	Enamel	4.3	2.0	-5.9	24.7	11	Average value of serial sampling
A7895	*Bos* sp.	M fragment	Enamel	2.7	3.2	-9.6	20.9	30	
A20	*Naemorhedus griseus*	m1	Enamel	4.9	-0.4	-2.8	28.0	2	Data from [17]
A258	*Naemorhedus griseus*	m2	Enamel	5.1	-4.3	-5.0	25.7	5	Data from [17]
A897	*Naemorhedus griseus*	m fragment	Enamel	5.8	-0.8	-6.9	23.7	7	Data from [17]
A973	*Naemorhedus griseus*	m3	Enamel	4.2	0.2	-5.6	25.1	8	Data from [17]
A1181	*Naemorhedus griseus*	M3	Enamel	5.4	0.6	-5.4	25.2	10	Data from [17]
A1301	*Naemorhedus griseus*	M fragment	Enamel	5.4	0.4	-8.9	21.7	11	Data from [17]
A1542	*Naemorhedus griseus*	m1	Enamel	6.9	-3.2	-4.6	26.1	12	Data from [17]
A1602	*Naemorhedus griseus*	M fragment	Enamel	5.7	-7.1	-0.3	30.6	14	Data from [17]
A1753	*Naemorhedus griseus*	M3	Enamel	4.6	0.2	-6.1	24.6	15	Data from [17]
A1805	*Naemorhedus griseus*	m1	Enamel	4.1	-3.0	-3.5	27.2	15	Data from [17]
A2006	*Naemorhedus griseus*	M3 fragment	Enamel	4.0	0.9	-6.7	23.9	16	Data from [17]
A2482	*Naemorhedus griseus*	m3 fragment	Enamel	3.4	1.9	-3.9	26.8	18	Data from [17]
A2998	*Naemorhedus griseus*	m3 fragment	Enamel	4.2	-0.3	-7.6	23.0	19	Data from [17]
A3439	*Naemorhedus griseus*	m2	Enamel	5.4	-3.0	-6.5	24.2	20	Data from [17]
A3441	*Naemorhedus griseus*	M1	Enamel	6.8	-2.9	-7.8	22.8	20	Data from [17]
A6713	*Naemorhedus griseus*	M3	Enamel	3.6	0.0	-4.1	26.6	25	Data from [17]
A7374	*Naemorhedus griseus*	m3	Enamel	3.8	0.5	-4.4	26.3	26	Data from [17]

A7377	*Naemorhedus griseus*	M2	Enamel	4.4	-0.6	-6.7	24.0	26	Data from [17]
A7846	*Naemorhedus griseus*	M2 fragment	Enamel	4.2	-3.7	-7.2	23.4	29	Data from [17]
A521	*Naemorhedus goral*	m2	Enamel	4.6	-1.0	-3.7	27.1	6	Data from [17]
A791	*Naemorhedus goral*	m1	Enamel	5.5	-4.0	-1.8	29.0	7	Data from [17]
A1921	*Naemorhedus goral*	M3	Enamel	4.3	-0.6	-7.3	23.3	16	Data from [17]
A1933	*Naemorhedus goral*	M2	Enamel	4.4	-2.5	-5.5	25.2	16	Data from [17]
A3707	*Naemorhedus goral*	m3	Enamel	3.4	-0.1	-11.4	19.1	21	Data from [17]
A7154	*Naemorhedus goral*	m2	Enamel	6.9	-5.3	-7.5	23.1	26	Data from [17]
A7375	*Naemorhedus goral*	M2	Enamel	5.3	-4.6	-2.8	28.0	26	Data from [17]
A7788	*Naemorhedus goral*	m3	Enamel	6.9	-4.5	-7.4	23.3	28	Data from [17]
A9	*Capricornis sumatraensis*	M2	Enamel	6.3	-2.7	0.1	30.9	1	Data from [17]
A530	*Capricornis sumatraensis*	p4	Enamel	4.5	-12.3	-7.9	22.7	6	Data from [17]
A738	*Capricornis sumatraensis*	M3	Enamel	6.0	-12.8	-5.6	25.1	7	Data from [17]
			Dentine	5.9	-11.5	-5.5	25.2	7	Data from [17]
A980	*Capricornis sumatraensis*	m2	Enamel	3.5	-11.5	-6.9	23.7	8	Data from [17]
A1431	*Capricornis sumatraensis*	m2	Enamel	3.8	-12.3	-8.1	22.5	12	Data from [17]
A1752	*Capricornis sumatraensis*	m1 fragment	Enamel	3.9	-13.2	-7.2	23.4	15	Data from [17]
			Dentine	6.9	-12.8	-5.8	24.9	15	Data from [17]
A4208	*Capricornis sumatraensis*	M2	Enamel	3.8	-14.0	-3.9	26.8	21	Data from [17]
A7362	*Capricornis sumatraensis*	M1	Enamel	3.8	-3.6	-4.4	26.3	26	Data from [17]
A7371	*Capricornis sumatraensis*	m2	Enamel	3.5	-12.0	-6.3	24.4	26	Data from [17]
A7663	*Capricornis sumatraensis*	m1	Enamel	6.3	-4.6	-6.4	24.3	27	Data from [17]
A7843	*Capricornis sumatraensis*	M3 fragment	Enamel	6.7	1.9	-7.8	22.8	29	Data from [17]
A7847	*Capricornis sumatraensis*	M2	Enamel	4.1	-14.3	-4.1	26.7	29	Data from [17]

Supplementary Table S5. Mann-Whitney pairwise comparisons of δ^{13}C and δ^{18}O values of tooth enamel for all mammalian taxa from Tham Lod Rockshelter.

**δ^{13}C (VPDB)**	***Macaca* sp. from TLR (n=8)**	***Homo sapiens* from TLR (n=5)**	***Hystrix* sp. From TLR (n=11)**	**Rodentia indet. from TLR (n=8)**	***Ursus thibetanus* from TLR (n=25)**	***Sus scrofa* from TLR (n=14)**	***Rucervus eldii* from TLR (n=11)**	***Rusa unicolor* from TLR (n=35)**	***Bubalus arnee* from TLR (n=5)**	***Bos gaurus* from TLR (n=13)**	***Naemorhedus griseus* from TLR (n=19)**	***Naemorhedus goral* from TLR (n=8)**	***Capricornis sumatraensis* from TLR (n=12)**
***Macaca* sp. from TLR (n=8)**	-	Z=-2.13; *p*=0.03	Z=2.28; *p*=0.02	Z=-2.58; *p*<0.01	Z=2.80; *p*<0.01	Z=2.84; *p*<0.01	Z=3.60; *p*<0.01	Z=3.73; *p*<0.01	Z=2.87; *p*<0.01	Z=3.74; *p*<0.01	Z=3.64; *p*<0.01	Z=2.79; *p*<0.01	Z=2.74; *p*<0.01
***Homo sapiens* from TLR (n=5)**		-	Z=2.10; *p*=0.04	Z=0.37; *p*=0.71	Z=2.17; *p*=0.03	Z=1.76; *p*=0.08	Z=3.07; *p*<0.01	Z=3.56; *p*<0.01	Z=-2.51; *p*=0.01	Z=3.16; *p*<0.01	Z=3.34; *p*<0.01	Z=2.85; *p*<0.01	Z=0.32; *p*=0.75
***Hystrix* sp. From TLR (n=11)**			-	Z=1.90; *p*=0.06	Z=0.69; *p*=0.49	Z=4.19; *p*<0.01	Z=3.94; *p*<0.01	Z=4.95; *p*<0.01	Z=3.06; *p*<0.01	Z=4.17; *p*<0.01	Z=4.48; *p*<0.01	Z=3.59; *p*<0.01	Z=2.03; *p*=0.04
**Rodentia indet. from TLR (n=8)**				-	Z=2.44; *p*=0.01	Z=1.40; *p*=0.16	Z=3.60; *p*<0.01	Z=3.45; *p*<0.01	Z=2.85; *p*<0.01	Z=3.66; *p*<0.01	Z=3.45; *p*<0.01	Z=2.57; *p*=0.01	Z=0.15; *p*=0.88
***Ursus thibetanus* from TLR (n=25)**					-	Z=4.73; *p*<0.01	Z=4.71; *p*<0.01	Z=6.37; *p*<0.01	Z=3.45; *p*<0.01	Z=4.99; *p*<0.01	Z=5.55; *p*<0.01	Z=4.01; *p*<0.01	Z=2.71; *p*<0.01
***Sus scrofa* from TLR (n=14)**						-	Z=2.71; *p*<0.01	Z=4.51; *p*<0.01	Z=3.19; p<0.01	Z=4.39; *p*<0.01	Z=4.41; *p*<0.01	Z=2.83; *p*<0.01	Z=1.67; *p*=0.09
***Rucervus eldii* from TLR (n=11)**							-	Z=4.89; *p*<0.01	Z=0.62; *p*=0.53	Z=2.81; *p*<0.01	Z=4.39; *p*<0.01	Z=3.60; *p*<0.01	Z=3.91; *p*<0.01
***Rusa unicolor* from TLR (n=35)**								-	Z=3.48; *p*<0.01	Z=4.54; *p*<0.01	Z=2.26; *p*=0.02	Z=0.39; *p*=0.70	Z=3.48; *p*<0.01
***Bubalus arnee* from TLR (n=5)**									-	Z=1.87; *p*=0.06	Z=2.99; *p*<0.01	Z=2.85; *p*<0.01	Z=3.01; *p*<0.01
***Bos gaurus* from TLR (n=13)**										-	Z=2.90; *p*<0.01	Z=3.44; *p*<0.01	Z=3.62; *p*<0.01
***Naemorhedus griseus* from TLR (n=19)**											-	Z=1.97; *p*=0.05	Z=3.41; *p*<0.01
***Naemorhedus goral* from TLR (n=8)**												-	Z=2.32; *p*=0.02
***Capricornis sumatraensis* from TLR (n=12)**													-
**δ^{18}O (VPDB)**	***Macaca* sp. from TLR (n=8)**	***Homo sapiens* from TLR (n=5)**	***Hystrix* sp. From TLR (n=11)**	**Rodentia indet. from TLR (n=8)**	***Ursus thibetanus* from TLR (n=25)**	***Sus scrofa* from TLR (n=14)**	***Rucervus eldii* from TLR (n=11)**	***Rusa unicolor* from TLR (n=35)**	***Bubalus arnee* from TLR (n=5)**	***Bos gaurus* from TLR (n=13)**	***Naemorhedus griseus* from TLR (n=19)**	***Naemorhedus goral* from TLR (n=8)**	***Capricornis sumatraensis* from TLR (n=12)**
***Macaca* sp. from TLR (n=8)**	-	Z=0.37; *p*=0.71	Z=0.83; *p*=0.41	Z=0.95; *p*=0.34	Z=0.13; *p*=0.90	Z=3.07; *p*<0.01	Z=0.04; *p*=0.97	Z=1.08; *p*=0.28	Z=0.07; *p*=0.94	Z=0.14; *p*=0.88	Z=0.50; *p*=0.61	Z=0.00; *p*=1.00	Z=0.04; *p*=0.97
***Homo sapiens* from TLR (n=5)**		-	Z=0.00; *p*=1.00	Z=0.44; *p*=0.66	Z=0.17; *p*=0.24	Z=2.50; *p*=0.01	Z=1.13; *p*=0.26	Z=0.29; *p*=0.77	Z=1.05; *p*=0.29	Z=0.94; *p*=0.35	Z=1.39; *p*=0.16	Z=0.22; *p*=0.83	Z=0.63; *p*=0.53
***Hystrix* sp. From TLR (n=11)**			-	Z=0.12; *p*=0.90	Z=1.36; *p*=0.17	Z=3.15; *p*<0.01	Z=1.51; *p*=0.13	Z=0.19; *p*=0.85	Z=1.08; *p*=0.28	Z=0.90; *p*=0.37	Z=1.87; *p*=0.06	Z=0.95; *p*=0.34	Z=1.08; *p*=0.28

**Rodentia indet. from TLR (n=8)**				-	Z=2.29; *p*=0.02	Z=2.80; *p*<0.01	Z=1.78; *p*=0.08	Z=0.20; *p*=0.84	Z=1.61; *p*=0.11	Z=1.52; *p*=0.13	Z=2.07; *p*=0.04	Z=0.89; *p*=0.37	Z=1.08; *p*=0.28
***Ursus thibetanus* from TLR (n=25)**					-	Z=3.94; *p*<0.01	Z=0.69; *p*=0.49	Z=1.49; *p*=0.14	Z=0.06; *p*=0.96	Z=0.23; *p*=0.82	Z=1.00; *p*=0.32	Z=0.02; *p*=0.98	Z=0.06; *p*=0.95
***Sus scrofa* from TLR (n=14)**						-	Z=3.53; *p*<0.01	Z=3.40; *p*<0.01	Z=2.64; *p*=0.01	Z=3.38; *p*<0.01	Z=4.08; *p*<0.01	Z=2.36; *p*=0.02	Z=3.53; *p*<0.01
***Rucervus eldii* from TLR (n=11)**							-	Z=1.57; *p*=0.12	Z=0.40; *p*=0.69	Z=0.35; *p*=0.73	Z=0.41; *p*=0.68	Z=0.12; *p*=0.90	Z=-0.12; *p*=0.90
***Rusa unicolor* from TLR (n=35)**								-	Z=0.96; *p*=0.34	Z=1.23; *p*=0.22	Z=2.26; *p*=0.02	Z=0.98; *p*=0.33	Z=1.37; *p*=0.17
***Bubalus arnee* from TLR (n=5)**									-	Z=1.15; *p*=0.88	Z=0.50; *p*=0.62	Z=0.07; *p*=0.94	Z=0.05; *p*=0.96
***Bos gaurus* from TLR (n=13)**										-	Z=0.71; *p*=0.48	Z=0.18; *p*=0.86	Z=0.05; *p*=0.96
***Naemorhedus griseus* from TLR (n=19)**											-	Z=0.27; *p*=0.79	Z=0.57; *p*=0.57
***Naemorhedus goral* from TLR (n=8)**												-	Z=0.16; *p*=0.91
***Capricornis sumatraensis* from TLR (n=12)**													-

Supplementary Table S6. Serial carbon and oxygen isotope values, calcium carbonate content, and relative distances from the cemento-enamel junction of the tooth crown (CEJ) of large bovids from the Area 1 of Tham Lod Rockshelter in Pang Mapha, northwestern Thailand.

Specimen no.	Taxon	Locality	Distance from CEJ (mm)	$CaCO_3$ (%)	$\delta^{13}C$ (‰VPDB)	$\delta^{18}O$ (‰VPDB)	$\delta^{18}O$ (‰VSMOW)
A920	*Bos javanicus*	Layer 7, Area 1, Tham Lod Rockshelter	2	4.5	1.9	-6.8	23.8
			6	3.9	1.6	-6.1	24.6
			10	4.2	1.7	-5.4	25.2
			14	4.1	1.6	-4.9	25.8
			18	4.4	1.6	-4.4	26.3
			22	4.3	1.4	-4.4	26.3
			26	4.1	1.4	-4.7	26.0
			30	4.2	1.1	-5.1	25.6
			34	4.2	1.2	-6.0	24.7
A1207	*Bos gaurus*	Layer 10, Area 1, Tham Lod Rockshelter	2	4.1	0.9	-8.2	22.4
			6	3.7	0.4	-8.6	22.0
			10	3.9	0.5	-8.5	22.1
			14	4.1	0.2	-8.9	21.7
			18	3.9	-0.2	-8.6	22.0
			22	4.6	-0.5	-8.5	22.1
			26	4.8	-1.0	-7.5	23.2
			30	4.5	-1.4	-7.0	23.6
			34	4.2	-2.1	-6.8	23.9
A1291	*Bos* sp.	Layer 11, Area 1, Tham Lod Rockshelter	2	4.9	1.7	-4.0	26.7
			6	4.5	1.8	-5.3	25.4
			10	4.2	2.1	-6.0	24.7
			14	3.7	2.1	-6.3	24.4
			18	4.4	1.9	-6.6	24.0
			22	4.4	1.9	-6.7	24.0
			26	4.6	2.0	-6.3	24.4
			30	4.1	2.2	-6.1	24.5
			34	3.9	2.7	-6.0	24.6
A1677	*Bos gaurus*	Layer 14, Area 1, Tham Lod Rockshelter	2	5.5	1.1	-5.4	25.3
			6	4.4	1.4	-5.0	25.7
			10	4.2	1.3	-4.5	26.2
			14	4.3	1.0	-4.1	26.6
			18	4.8	0.8	-4.3	26.4
			22	4.5	0.9	-4.3	26.4
			26	4.8	0.6	-5.9	24.8

			30	4.5	0.6	-6.4	24.3	
			34	4.6	0.5	-6.5	24.1	
A3697	*Bos gaurus*	Layer 21, Area 1, Tham Lod Rockshelter	2	3.5	0.8	-6.6	24.1	
			7	3.4	0.3	-7.1	23.5	
			12	3.5	0.1	-7.3	23.3	
			17	3.9	-0.3	-7.9	22.7	
			22	3.6	-0.1	-8.2	22.4	
			27	3.9	-0.1	-8.8	21.8	
			32	3.8	-0.1	-8.4	22.2	
			37	3.6	-0.3	-8.6	21.9	
			42	3.6	-0.4	-8.6	22.0	

Supplementary Table S7. A stable carbon isotope dataset of extant mammal tooth enamel from mainland Southeast Asia. A Suess effect δ^{13}C correction is applied for samples of modern mammals that died after ad 1950 ([18]; [19]). Collection abbreviations: **AMNH**=Mammalogy collection, American Museum of Natural History (New York, USA); **MNHN ZM-MO**=Zoological collection of mammals and birds, Muséum National d'Histoire Naturelle (Paris, France); **CU-MM-T**=Thai mammal collection, Chulalongkorn University Museum of Natural History (Bangkok, Thailand); **THNHM-M**=Mammal collection, Thailand Natural History Museum (Pathum Thani, Thailand); **ZSM**=Zoologische Staatssammlung München (Munich, Germany); **ZRC**= Zoological Reference Collection, Lee Kong Chian Natural History Museum (Singapore).

**Specimen no.**	**Species/taxon**	**Dietary type**	**Tissue/tooth**	**Locality**	**Year of death/animal collection**	**δ^{13}C (‰VPDB)**	**Suess corrections to δ^{13}C**	**References**
**Primates**								
MNHN ZM-MO-CG-1882-1	*Macaca fasicularis*	Omnivore	m3	Poulo Condor, Vietnam	1882	-11.0		[20]
MNHN ZM-MO-CG-11878-1126	*Macaca nemestrina*	Omnivore	m3	Chamchay, Chochinchine, Vietnam	1878	-15.2		[20]
MNHN ZM-MO-CG-1877-760	*Macaca nemestrina*	Omnivore	M3	30km from Kuala Lumpar, Forest, Malaysia	1977	-15.1	-14.1	[20]
Unnumbered	*Macaca nemestrina*	Omnivore	Enamel	Singapore	Unknown	-16.8		[21]
Unnumbered	*Macaca nemestrina*	Omnivore	Enamel	Singapore	Unknown	-16.7		[21]
Unnumbered	*Macaca nemestrina*	Omnivore	Enamel	Singapore	Unknown	-16.3		[21]
MNHN ZM-MO-CG-1892-1357	*Macaca mulatta*	Omnivore	m2	Nam-Ou 20, 20 N, 102, 15E, Laos	1892	-13.7		[20]
MNHN ZM-MO-CG-1899-54	*Macaca mulatta*	Omnivore	m3	Song-Ta-Voy, near Huong Binh and Huong-Him, Annam, Vietnam	1899	-15.3		[20]
MNHN ZM-MO-CG-1935-201	*Macaca arctoides*	Omnivore	m2	Cochinchine, Vietnam?	1935	-12.9		[20]
MNHN ZM-MO-CG-2009-307	*Presbytis siamensis*	Herbivore	m3	Indochina	1948	-14.4		[20]
MNHN ZM-MO-CG-1877-695/A-12492	*Pygathrix nemaeus*	Herbivore	m3	Unknown	1877	-14.4		[20]
MNHN ZM-MO-CG-1897-518/519	*Pygathrix nemaeus*	Herbivore	m3	Vietnam	1897	-14.5		[20]
MNHN ZM-MO-CG-1929-442	*Pygathrix nemaeus*	Herbivore	m3	Hafe, Laos	1928	-14.7		[20]
MNHN ZM-MO-A-3845	*Pygathrix nemaeus*	Herbivore	m3	Unknown	Unknown	-16.0		[20]
MNHN ZM-MO-CG-1878-1123	*Pygathrix nigripes*	Herbivore	m3	Vietnam	1878	-14.8		[20]
MNHN ZM-MO-CG-1886-1122	*Pygathrix nigripes*	Herbivore	m3	Saigon, Vietnam	1886	-14.4		[20]
MNHN ZM-MO-CG-1929-444	*Pygathrix nigripes*	Herbivore	m3	Djering vers 1000m, sud de l'Annam, Vietnam	1927	-16.1		[20]

MNHN ZM-MO-CG-1929-448	*Rhinopithecus avunculus*	Herbivore	m3	Aoupa, Tonkin, Vietnam	1929	-15.8		[20]
MNHN ZM-MO-CG-1929-449	*Rhinopithecus avunculus*	Herbivore	m3	Bac Ran Tonkin 500 ft, Vietnam	1927	-15.9		[20]
MNHN ZM-MO-CG-1929-450	*Rhinopithecus avunculus*	Herbivore	m3	Bac Ran Tonkin 500 ft, Vietnam	1927	-16.5		[20]
MNHN ZM-MO-CG-1929-457	*Trachypithecus barbei*	Herbivore	m3	Saigon, Vietnam	1929	-16.7		[20]
MNHN ZM-MO-CG-1878-1124	*Trachypithecus cristatus*	Herbivore	m3	Ile de Phu Koc, Vietnam	1878	-15.8		[20]
MNHN ZM-MO-CG-2001-112	*Trachypithecus cristatus*	Herbivore	m3	Selangon, Peninsular Malaysia	1976	-15.1	-14.1	[20]
MNHN ZM-MO-1929-440	*Trachypithecus francoisi*	Herbivore	m3	Saigon, Vietnam	1926	-15.9		[20]
MNHN ZM-MO-CG-1929-439	*Trachypithecus francoisi*	Herbivore	m2	Bac Ran Tonkin, Vietnam	1927	-16.4		[20]
MNHN ZM-MO-CG-1899-51	*Trachypithecus germaini*	Herbivore	m3	Nha Trang Aunam, Vietnam	1899	-15.9		[20]
MNHN ZM-MO-CG-1899-53	*Trachypithecus germaini*	Herbivore	m3	Nha Trang Aunam, Vietnam	1899	-15.6		[20]
MNHN ZM-MO-CG-1962-1382	*Trachypithecus germaini*	Herbivore	M3	Vietnam	1926	-16.0		[20]
MNHN ZM-MO-CG-1882-2925	*Trachypithecus obscurus*	Herbivore	M2	Thailand	1882	-16.7		[20]
MNHN ZM-MO-CG-1929-436	*Trachypithecus phayrei*	Herbivore	m3	Nghia Hung Nord de l'Annam, Vietnam	1929	-15.3		[20]
MNHN ZM-MO-CG-1929-437	*Trachypithecus phayrei*	Herbivore	m3	Phu Qui Annam, Vietnam	1928	-16.0		[20]
MNHN ZM-MO-CG-1934-546	*Trachypithecus phayrei*	Herbivore	m3	Naynei? Son?	1934	-15.6		[20]
Unnumbered	*Trachypithecus phayrei*	Herbivore	Enamel	Singapore	Unknown	-16.4		[21]
MNHN ZM-MO-CG-1962-1488	*Nomascus concolor concolor*	Omnivore	m3	Tonkin, Vietnam	1897	-14.4		[20]
MNHN ZM-MO-CG-1929-451	*Nomascus gabriellae*	Omnivore	m2	Dalat, Annam, Vietnam	1929	-14.4		[20]
MNHN ZM-MO-CG-1964-1616	*Nomascus gabriellae*	Omnivore	m2	Menagerie, Indochina	1963	-14.5	-13.8	[20]
MNHN ZM-MO-CG-1929-452	*Nomascus leucogenys*	Omnivore	m2	Xieng Quang, Laos, Indochina	1926	-15.1		[20]
MNHN ZM-MO-CG=1942=176	*Nomascus leucogenys*	Omnivore	m3	Laos, Indochina	1942	-14.2		[20]
MNHN ZM-MO-CG-1962-1487	*Nomascus leucogenys*	Omnivore	m1	Indochina	1930	-12.1		[20]
MNHN ZM-MO-ZAC-2009-414	*Nomascus leucogenys*	Omnivore	m3	Tonkin, Vietnam	1840	-15.0		[20]
Unnumbered	*Hylobates lar*	Omnivore	Enamel	Thailand	Unknown	-16.0		[21]
MNHN ZM-MO-CG-1878-1122	*Hylobates pileatus*	Omnivore	m3	Cambodia	1878	-13.6		[20]
MNHN ZM-MO-CG-1896-275	*Hylobates pileatus*	Omnivore	m2	Thailand	1896	-14.4		[20]
MNHN ZM-MO-CG-1976-329	*Hylobates pileatus*	Omnivore	m1	Siem Reap, Cambodia	1969	-14.8	-14.0	[20]
**Rodentia**								
ZSM-1906/105	*Hystrix brachyura*	Omnivore	p4	Nong Nam Sai, Bangkok, Thailand	1906	-14.1		[22]

THNHM-M-08083	*Hystrix brachyura*	Omnivore	p4	Thailand	1980	-16.3	-15.3	[22]
THNHM-M-02420	*Hystrix brachyura*	Omnivore	i1	Ban Phu Toei, Sai Yok, Kanchanaburi, Thailand	1971	-14.2	-13.4	[22]
Unnumbered	*Hystrix* sp.	Omnivore	Enamel	Thung Yai, Thailand	Unknown	-18.6		[21]
**Carnivora**								
AMNH-112978	*Helarctos malayanus*	Omnivore	Enamel	Malay Peninsula, Perak	Unknown	-16.3		[20]
MNHN ZM-MO-CG-1914-360	*Helarctos malayanus*	Omnivore	m2	Ouis de Cochchine, Vietnam	1914	-13.0		[20]
MNHN ZM-MO-CG-1929-430	*Helarctos malayanus*	Omnivore	M2	Reg de Mai, Lanli, Annam, Vietnam	1929	-14.1		[20]
AMNH-114544	*Ursus thibetanus*	Omnivore	Enamel	Burma: Hpawshi hka 7400 ft	1989	-14.2	-12.9	[20]
MNHN ZM-MO-CG-1986-345	*Ursus thibetanus*	Omnivore	m2	Annam, Vientam	1938	-6.7		[20]
AMNH-299	*Arctictis binturong*	Omnivore	Enamel	Peninsular Malaysia, Melaka state, Melaka	Unknown	-16.2		[20]
MNHN ZM-MO-CG-1871	*Arctictis binturong*	Omnivore	C1	Malacca, Peninsular Malaysia	1871	-16.4		[20]
Unnumbered	*Viverra zibetha*	Omnivore	Enamel	Singapore	Unknown	-12.3		[21]
AMNH-87350	*Panthera pardus*	Carnivore	Enamel	Laos, Plateau Bolovens	Unknown	-13.1		[20]
MNHN ZM-MO-CG-1931-858	*Panthera pardus*	Carnivore	m2	Indochine, Vietnam?	1931	-13.0		[20]
AMNH-201798	*Panthera tigris*	Carnivore	Enamel	Jelai River, Pahang, Peninsular Malaysia	1931	-13.8		[20]
Unnumbered	*Panthera tigris*	Carnivore	Enamel	Singapore	Unknown	-8.7		[21]
Unnumbered	*Felis* sp.	Carnivore	Enamel	Thailand	Unknown	-15.2		[21]
**Proboscidea**								
ZRC4.1670	*Elephas maximus*	Herbivore	Molar	Unknown	Unknown	-16.4		[20]
ZRC4.1673	*Elephas maximus*	Herbivore	Molar	Unknown	Unknown	-18.0		[20]
ZRC4.7880	*Elephas maximus*	Herbivore	Molar	Unknown	1877	-12.8		[20]
ZRC4.7881	*Elephas maximus*	Herbivore	Molar	Perak, Chenor, Peninsular Malaysia, found on the property of Wannli Hydraulic Tin Mines	1936	-12.5		[20]
ZRC4.7882	*Elephas maximus*	Herbivore	Molar	Perak, Kampor, Peninsular Malaysia. Found associated with Neolithic adze	Unknown	-12.8		[20]
ZRC4.7883	*Elephas maximus*	Herbivore	Molar	Perak, Kampor, Peninsular Malaysia. Found associated with Neolithic adze	Unknown	-12.4		[20]
ZRC4.7884	*Elephas maximus*	Herbivore	Molar	Johore, Peninsular Malaysia, Dug out near Muar River	1908	-12.8		[20]

ZRC4.7885	*Elephas maximus*	Herbivore	Molar	Johore, Peninsular Malaysia, Dug out near Muar River	1908	-12.6		[20]
Unnumbered	*Elephas maximus*	Herbivore	Enamel	Huai Kha Khaeng, Thailand	Unknown	-11.4		[21]
Unnumbered	*Elephas maximus*	Herbivore	Enamel	Khao Soi Dao, Thailand	Unknown	-18.4		[21]
**Perissodactyla**								
THNHM-M-00192-1	*Rhinoceros sondaicus*	Herbivore	P4	Thailand	≈1950	-5.9		[22]
THNHM-M-00192-2	*Rhinoceros sondaicus*	Herbivore	M2	Thailand	≈1950	-11.9		[22]
THNHM-M-00192-2*	*Rhinoceros sondaicus*	Herbivore	M2	Thailand	≈1950	-12.3		[22]
Unnumbered	*Rhinoceros sondaicus*	Herbivore	Enamel	Kanchanaburi, Thailand	Unknown	-18.0		[21]
AMNH-54763	*Dicerorhinus sumatrensis*	Herbivore	Enamel	Burma	1924	-14.6		[20]
AMNH-81892	*Dicerorhinus sumatrensis*	Herbivore	Enamel	Malaysia	Unknown	-18.0		[20]
**Artiodactyla**								
ZRC4.1835	*Sus verruscosus*	Omnivore	M2	Shanghai Klebang Est. nr. Ipoh, Perak, Malaysia	Unknown	-13.9		[20]
ZRC4.4750	*Tragulus napu*	Herbivore	M2	Pulau Ubin, Singapore	1921	-13.8		[20]
ZRC4.4753	*Tragulus napu*	Herbivore	m2	Bandon (Surat Thani), Thailand	1931	-19.4		[20]
ZRC4.4789	*Tragulus napu*	Herbivore	M3	Thailand: Pulau Terutau, Telok Wau	1916	-18.1		[20]
MNHN ZM-MO-1971-72	*Axis porcinus*	Herbivore	m3	Cochinchine, Vietnam	1932	-5.4		[20]
ZRC4.1737	*Muntiacus muntjak*	Herbivore	m2	Ulu Gombak, Selangor, Peninsular Malaysia	1916	-13.9		[20]
ZRC4.1738	*Muntiacus muntjak*	Herbivore	M3	Bukit Tangga, Negri Sembilan, Peninsular Malaysia	1914	-14.7		[20]
ZRC4.1762	*Muntiacus muntjak*	Herbivore	M2	Hat Sanuh, nr. Koh Lak, Rajburi, SW Thailand	1919	-11.9		[20]
ZRC4.1765	*Muntiacus muntjak*	Herbivore	M2	Krian Rd, Taiping, Perak, Peninsular Malaysia	1910	-15.0		[20]
MNHN ZM-MO-1932-76	*Muntiacus muntjak*	Herbivore	m3	Indochina	1932	-12.6		[20]
THNHM-M-07272	*Rusa unicolor*	Herbivore	M2	Thailand	≈1950	-15.8		[22]
THNHM-M-00124	*Rusa unicolor*	Herbivore	M3	Thailand	≈1950	-14.7		[22]
THNHM-M-07273	*Rusa unicolor*	Herbivore	M3	Thailand	≈1950	-14.8		[22]
THNHM-M-08060	*Rusa unicolor*	Herbivore	M3	Thailand	≈1950	-16.2		[22]
THNHM-M-00193	*Rusa unicolor*	Herbivore	M2	Thailand	≈1950	-13.3		[22]
MNHN ZM-MO-1874-190	*Rusa unicolor*	Herbivore	m2	Vietnam	1878	-14.1		[20]
Unnumbered	*Rusa unicolor*	Herbivore	Enamel	Huai Kha Khaeng, Thailand	Unknown	-3.0		[21]
MNHN ZM-MO-1932-179	*Cervus* sp./*Rusa* sp.	Herbivore	m2	Residence de Pusat, Cambodia	1932	-3.8		[20]

ZRC4.1800	*Cervus* sp./*Rusa* sp.	Herbivore	m3	Johore, Peninsular Malaysia	Unknown	-15.0		[20]
Unnumbered	*Cervus* sp./*Rusa* sp.	Herbivore	Enamel	Thailand	Unknown	-8.0		[21]
AMNH-54583	*Bos javanicus*	Herbivore	Enamel	Indochina	Unknown	-3.1		[20]
AMNH-113755	*Bos javanicus*	Herbivore	Enamel	Annan, Dong Me, Vietnam	1936	-7.5		[20]
AMNH-113756	*Bos javanicus*	Herbivore	Enamel	Annan, Dong Me, Vietnam	1936	1.1		[20]
AMNH-113758	*Bos javanicus*	Herbivore	Enamel	Cochinchine, Lagua River, Vietnam	1936	-3.1		[20]
AMNH-54582	*Bos sauveli*	Herbivore	Enamel	Indo-China	Unknown	1.3		[20]
AMNH-87621	*Bos sauveli*	Herbivore	Enamel	Unknown	Unknown	-6.5		[20]
MNHN ZM-MO-1877-651	*Bos gaurus*	Herbivore	P2?	Cochinchine, Vietnam	1877	-2.5		[20]
Unnumbered	*Bos gaurus*	Herbivore	Enamel	Thailand	Unknown	-5.9		[21]
THNHM-M-00159	*Capricornis sumatraensis*	Herbivore	M3	Thailand	≈1950	-8.8		[17]
THNHM-M-07275	*Capricornis sumatraensis*	Herbivore	M3	Thailand	≈1950	-14.3		[17]
THNHM-M-07276	*Capricornis sumatraensis*	Herbivore	M3	Thailand	≈1950	-9.7		[17]
THNHM-M-08004	*Capricornis sumatraensis*	Herbivore	M3	Thailand	≈1950	-15.5		[17]
THNHM-M-07971	*Capricornis sumatraensis*	Herbivore	M3	Thailand	≈1950	-13.6		[17]
THNHM-M-07969	*Capricornis sumatraensis*	Herbivore	M3	Thailand	≈1950	-12.4		[17]
THNHM-M-07277	*Capricornis sumatraensis*	Herbivore	M3	Thailand	≈1950	-14.6		[17]
CU-MM-T-0002	*Capricornis sumatraensis*	Herbivore	M3	Thailand	≈1950	-11.9		[17]
CU-MM-T-0180	*Capricornis sumatraensis*	Herbivore	M3	Thailand	≈1950	-12.0		[17]
CU-MM-T-0181	*Capricornis sumatraensis*	Herbivore	M3	Thailand	≈1950	-13.4		[17]
ZRC4.1685	*Capricornis sumatraensis*	Herbivore	M2	Muang Prae (Meh Lem) North Thailand	1916	-13.4		[20]
ZRC4.1688	*Capricornis sumatraensis*	Herbivore	M2	Koh Lok, SW Thailand	1905	-11.3		[20]
ZRC4.1691	*Capricornis sumatraensis*	Herbivore	M2	Bangkok, Thailand	Unknown	-12.2		[20]
MNHN ZM-MO-1993-4240	*Capricornis sumatraensis*	Herbivore	M3	Montagne kanouane, Savane, Laos (Alt. 600 m)	1933	-15.2		[20]
Unnumbered	*Capricornis sumatraensis*	Herbivore	Enamel	Laos	Unknown	-15.9		[21]
CU-MM-T-0182	*Naemorhedus griseus*	Herbivore	M3	Northern Thailand	≈1950	-3.3		[17]

Supplementary Table S8. $\delta^{13}C$ and $\delta^{18}O$ values and calcium carbonate content of soil carbonates from the Area 1 of Tham Lod Rockshelter in Pang Mapha, northwestern Thailand.

**Specimen no.**	**Locality**	**$CaCO_3$ (%)**	**$\delta^{13}C$ (‰VPDB)**	**$\delta^{18}O$ (‰VPDB)**	**$\delta^{18}O$ (‰VSMOW)**	**Layers**
TL-A1-276	Tham Lod Rockshelter (Area 1)	40.8	-17.6	-11.3	19.2	3
TL-A1-275	Tham Lod Rockshelter (Area 1)	35.1	-15.3	-10.2	20.4	14
TL-A1-277	Tham Lod Rockshelter (Area 1)	22.0	-15.2	-9.7	20.8	22
TL-A1-284	Tham Lod Rockshelter (Area 1)	23.9	-20.3	-13.7	16.7	23
TL-A1-281	Tham Lod Rockshelter (Area 1)	31.9	-13.2	-8.1	22.6	29

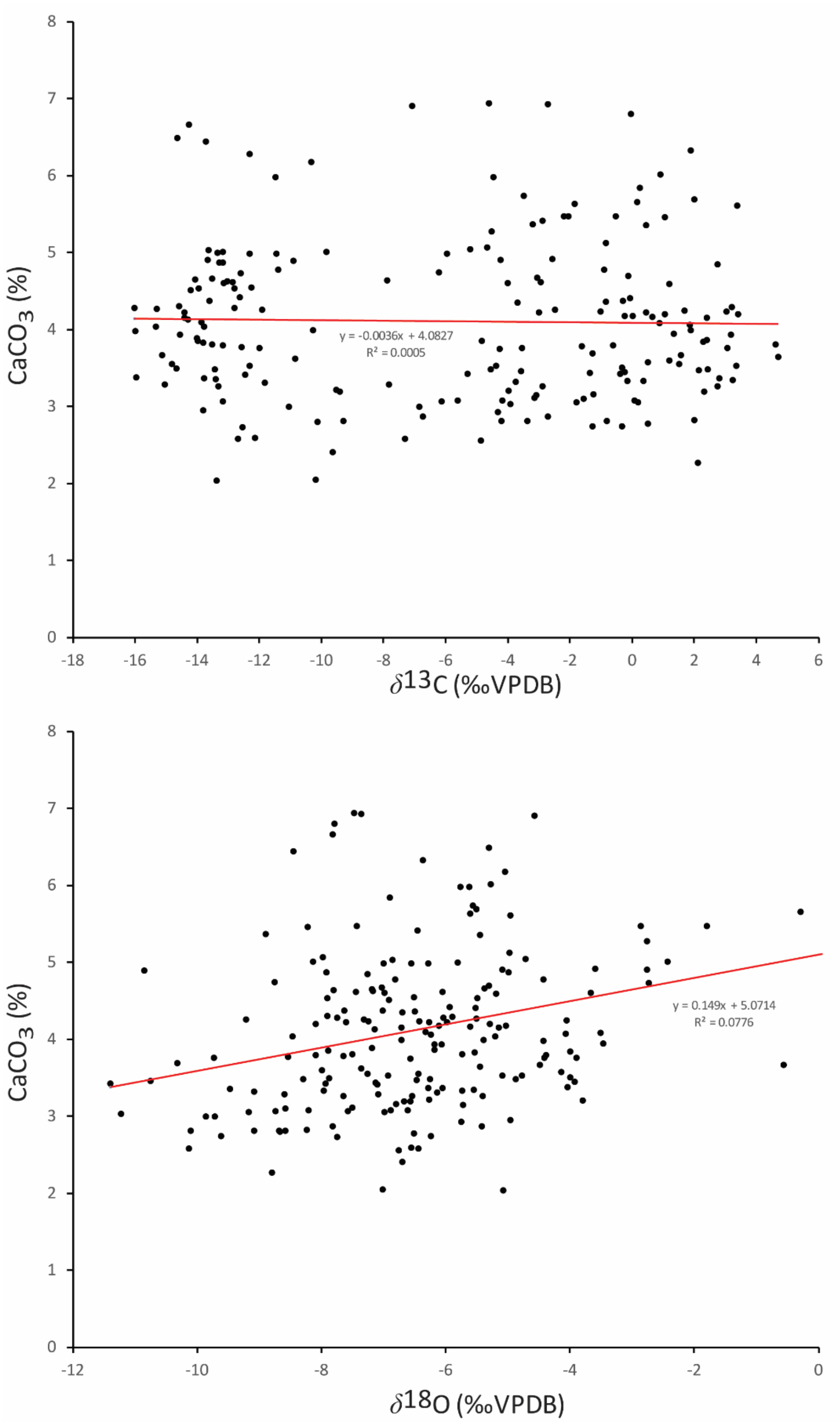

Supplementary Figure S1. Scatter diagrams of calcium carbonate content versus stable carbon and oxygen isotope values of samples from Tham Lod Rockshelter in highland Pang Mapha, northwestern Thailand.

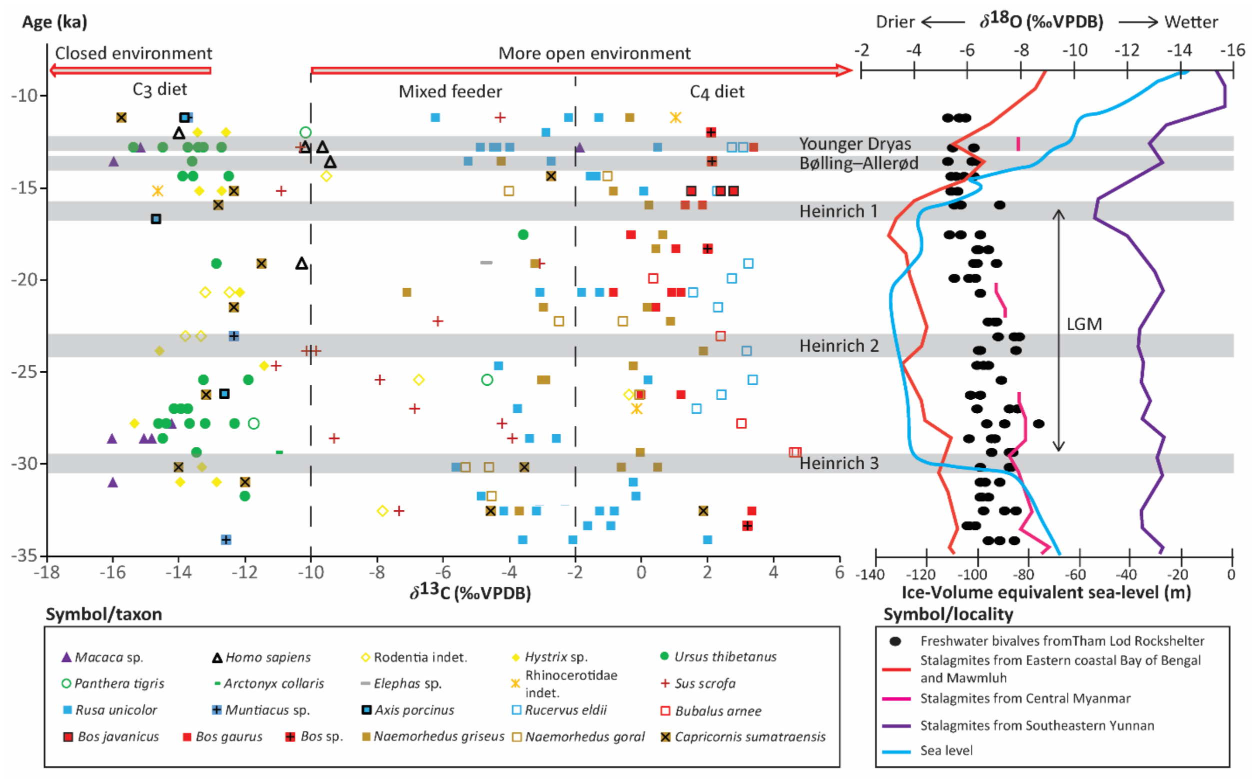

Supplementary Figure S2. Bulk $\delta^{13}C$ values of human and mammal tooth enamel through the stratigraphic sequence of Tham Lod Rockshelter. All isotope samples are plotted together in comparison with freshwater bivalve $\delta^{18}O$ data (black dot) collected from the same stratigraphic section (west profile) ([9]), with speleothem $\delta^{18}O$ records data using 1000-year averages from mainland Southeast Asia including eastern coastal Bay of Bengal and Mawmluh (red line), central Myanmar (pink line), and southeastern Yunnan (purple line) ([23]; [24]; [25]), and with sea level fluctuations (sky blue) ([26]) over the past 35 ka. High-resolution chronological data of the sequence follow the radiobarbon dates on freshwater bivalves collected from each stratigraphic layer ([9]).

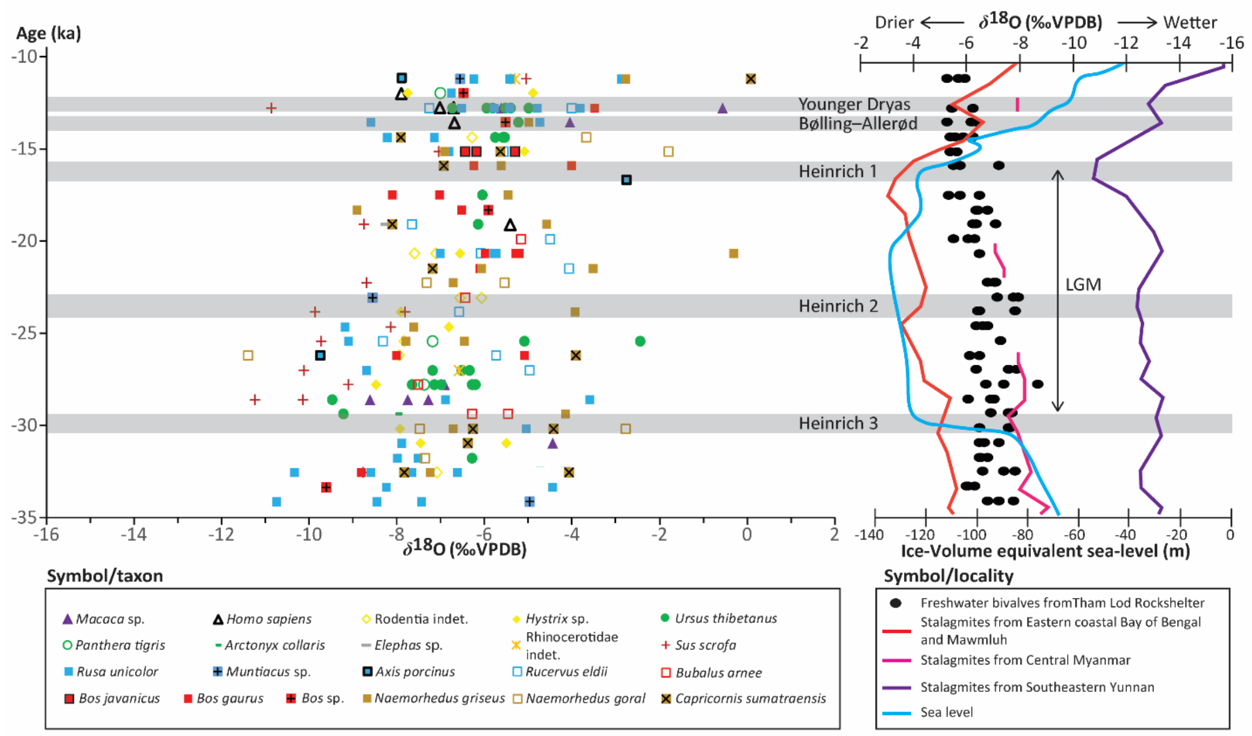

Supplementary Figure S3. Bulk δ^{18}O values of human and mammal tooth enamel through the stratigraphic sequence of Tham Lod Rockshelter. All isotope samples are plotted together in comparison with freshwater bivalve δ^{18}O data (black dot) collected from the same stratigraphic section (west profile) ([9]), with speleothem δ^{18}O records data using 1000-year averages from mainland Southeast Asia including eastern coastal Bay of Bengal and Mawmluh (red line), central Myanmar (pink line), and southeastern Yunnan (purple line) ([23]; [24]; [25]), and with sea level fluctuations (sky blue) ([26]) over the past 35 ka. High-resolution chronological data of the sequence follow the radiobarbon dates on freshwater bivalves collected from each stratigraphic layer ([9]).

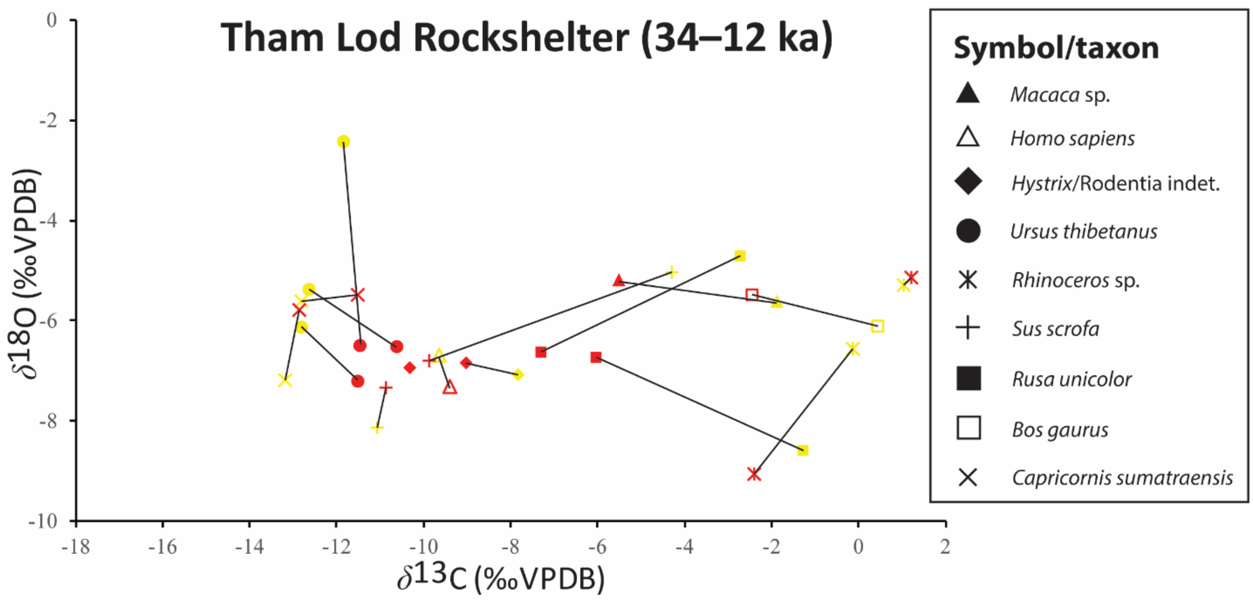

Supplementary Figure S4. A bivariate plot of stable carbon and oxygen isotope values of tooth enamel (yellow) and dentine (red) within the same individuals of humans and mammals from the late Pleistocene of Tham Lod Rockshelter in highland Pang Mapha, northwestern Thailand.

Supplementary information references

1) Kiernan, K., Spies, J., & Dunkley, J. Prehistoric occupation and burial sites in the mountains of the Nam Khong area, Mae Hongson province, northwestern Thailand. *Aust. Archaeol.* **27**, 24-44 (1988).

2) Smittinan, T. *Thai Plant Names (Botanical names-Vemacular names* (Royal Forest Department, 1980).

3) Smittinan, T., Santisuk, T. & Phengklai, C. *The Manual of Dipterocarpaceae of Mainland South-East Asia* (Forest Herbarium, 1980).

4) Shoocongdej, R. *et al. Final report of Highland Archaeology Project in Pang Mapha District, Mae Hong Son Province Phase 2, Vol. 2* (Thailand Research Fund, 2007).

5) Khaokhiew, C. *Geoarchaeology of Tham Lod Rockshelter, Changwat Mae Hong Son, Northern Thailand* (Chulalongkorn University, 2004).

6) Shoocongdej, R. & Wattanapituksakul, A. Faunal assemblages and demography during the Late Pleistocene (MIS 2-1) to Early Holocene in Highland Pang Mapha, Northwest Thailand. *Quat. Int.* **563**, 51–63 (2020).

7) Shoocongdej, R. Late Pleistocene activities at the Tham Lod rockshelter in Highland Pang Mapha, Mae Hong Son province, Norhwestern Thailand in *Uncovering Southeast Asia's Past* (eds. Bacus, E., Glover, I. & Pigott, V.) 22–37 (NUS Press, 2006).

8) Shoocongdej, R. Paleoenvironment during Late Pleistocene to Late Holocene on highland in Pang Mapha distinct, Mae Hong Son province in *Proceedings of the Conference on People, Culture, and Paleoenvironment in Highland Pang Mapha, Mae Hong Son Province* (ed. Shoocongdej, R.) 292–317 (Silpakorn University, 2003).

9) Marwick, B. & Gagan, M. K. Late Pleistocene monsoon variability in northwest Thailand: an oxygen isotope sequence from the bivalve *Margaritanopsis laosensis* excavated in Mae Hong Son province. *Quat. Sci. Rev.* **30**, 3088–3098 (2011).

10) Marwick, B. Multiple Optima in Hoabinhian flaked stone artefact palaeoeconomics and palaeoecology at two archaeological sites in Northwest Thailand. *J. Anthropol. Archaeol.* **32**, 553–564 (2013).

11) Wright, L. E. & Schwarcz, H. P. Correspondence between stable carbon, oxygen and nitrogen isotopes in human tooth enamel and dentine: infant diets at Kaminaljuyú. *J. Archaeol. Sci.* **26**, 1159–1170 (1999).

12) Bocherens, H., Fizet, M., & Mariotti, A. Diet, physiology and ecology of fossil mammals as inferred from stable carbon and nitrogen isotope biogeochemistry: implications for Pleistocene bears. *Palaeogeogr. Palaeoclimat. Palaeoecol.* **107**, 213–225 (1994).

13) Koch, P. L., Tuross, N. & Fogel, M. L. The effects of sample treatment and diagenesis on the isotopic integrity of carbonate in biogenic hydroxylapatite. *J. Archaeol. Sci.* **24**, 417–429 (1997).

14) Bocherens, H., Drucker, D., Billiou, D. & Moussa, I. Une nouvelle approche pour évaluer l'état de conservation de l'os et du collagène pour les mesures isotopiques (datation au radiocarbone, isotopes stables du carbone et de l'azote). *l'Anthropologie* **109**, 557-567 (2005).

15) Ambrose, S. H. Preparation and characterization of bone and tooth collagen for isotopic analysis. *J. Archeol. Sci.* **17**, 431–451 (1990).

16) van Klinken, G. J. Bone Collagen Quality Indicators for Palaeodietary and Radiocarbon Measurements. *J. Archaeol. Sci.* **26**, 687–695 (1999).

17) Suraprasit, K. *et al.* Long-Term Isotope Evidence on the Diet and Habitat Breadth of Pleistocene to Holocene Caprines in Thailand: Implications for the Extirpation and Conservation of Himalayan Gorals. *Front. Ecol. Evol.* **8**, 67; 10.3389/fevo.2020.00067 (2020).

18) Cerling, T. E. & Harris, J. M. Carbon isotope fractionation between diet and bioapatite in ungulate mammals and implications for ecological and paleoecological studies. *Oecologia* **120**, 347–363 (1999).

19) Passey, B.H. *et al.* Carbon isotope fractionation between diet, breath CO_2, and bioapatite in different mammals. *J. Archaeol.Sci.* **32**, 1459–1470 (2005).

20) Louys, J. & Roberts, P. Environmental drivers of megafauna and hominin extinction in Southeast Asia. *Nature* **586**, 402–406 (2020).

21) Pushkina, D., Bocherens, H., Chaimanee, Y. & Jaeger, J.-J. Stable carbon isotope reconstructions of diet and paleoenvironment from the late Middle Pleistocene Snake Cave in Northeastern Thailand. *Naturwissenschaften* **97**, 299–309 (2010).

22) Suraprasit, K., Jongautchariyakul, S., Yamee, C., Pothichaiya, C. & Bocherens, H. New fossil and isotope evidence for the Pleistocene zoogeographic transition and hypothesized savanna corridor in peninsular Thailand. *Quat. Sci. Rev.* **221**, 105861 (2019).

23) Dutt, S. *et al.* Abrupt changes in Indian summer monsoon strength during 33,800 to 5500 years B.P. *Geophys. Res. Lett.* **42**, 5526–5532 (2015).

24) Ronay, E. R., Breitenbach, S. F. M. & Oster, J. L. Sensitivity of speleothem records in the Indian Summer Monsoon region to dry season infiltration. *Sci. Rep.* **9**, 5091; 10.1038/s41598-019-41630-2 (2019).

25) Liu, G. *et al.* On the glacial-interglacial variability of the Asian monsoon in speleothem δ^{18}O records. *Sci. Adv.* **6**, eaay8189; 10.1126/sciadv.aay8189 (2020).

26) Lambeck, K., Rouby, H., Purcell, A., Sun, Y. & Sambridge, M. Sea level and global ice volumes from the Last Glacial Maximum to the Holocene. *Proc. Natl. Acad. Sci. U.S.A.* **111**, 15296–15303 (2014).
